# Dementia risk across distinct metabolic profiles in the UK Biobank

**DOI:** 10.1007/s11357-025-01970-6

**Published:** 2025-11-28

**Authors:** Amanda L. Lumsden, Anwar Mulugeta, Elina Hyppönen

**Affiliations:** 1 Australian Centre for Precision Health, Unit of Clinical and Health Sciences, University of South Australia, South Australian Health and Medical Research Institute, P.O. Box 11060, Adelaide, South Australia 5001 Australia; 2 South Australian Health and Medical Research Institute, North Terrace, Adelaide, South Australia 5001 Australia; 3 Department of Pharmacology and Clinical Pharmacy, College of Health Sciences, Addis Ababa University, Addis Ababa, Ethiopia

**Keywords:** Dementia risk, Metabolic subgroup, Metabolic profile, Biomarkers, Self-organising map

## Abstract

Sub-optimal metabolism is linked to dementia risk, yet metabolic traits rarely occur in isolation. Using data from 308,019 UK Biobank participants, we examined associations of six diverse metabolic subgroups (I–VI)** –** previously derived via a self-organising map (SOM) that captures patterns of co-occurring metabolic biomarker traits in the population** –** and 39 individual biomarkers, with incident all-cause dementia, Alzheimer’s disease (AD), and vascular dementia (VaD). Biomarker associations were assessed using both linear and nonlinear (restricted cubic spline) models. After adjusting for age, sex, socioeconomic, and lifestyle factors, subgroup analyses showed that participants in the two leanest and two most adipose subgroups had higher risk of dementia outcomes compared to others. Subgroups with high adiposity exhibited elevated VaD risk, which was linked to hypertension, hyperglycaemia, and liver stress (Subgroup II); inflammation, microalbuminuria, and low apolipoprotein A1 (III). For AD, the risk was elevated in the lean subgroups (IV, V), characterised by low body mass index (BMI), triglycerides, and urate, and high sex-hormone binding globulin; as well as for adipose Subgroup II. *APOE*-ε4 allele count had limited influence on dementia associations with metabolic subgroups and biomarkers. This marked metabolic heterogeneity in dementia risk suggests that metabolic profiling could inform targeted prevention strategies. Interpretation of these findings is supported by previously reported MRI profiles of the metabolic subgroups, providing biological context.

**Supplementary Information:**

The online version contains supplementary material available at https://doi.org/10.1007/s11357-025-01970-6.

## Introduction

Dementia is a heterogeneous group of neurodegenerative diseases affecting cognition and memory. There is evidence that sub-optimum metabolic states and conditions related to metabolic disturbances may alter the risk of developing dementia in later life, with past studies reporting links with metabolic syndrome [1], diabetes [2], fatty liver disease [3], and chronic kidney disease [4]. Dementia risk has also been associated with hormone balance [5–7], adiposity [8], and cardiovascular health [9]. Many aspects of metabolism are modifiable by lifestyle, nutrition, and use of pharmaceuticals, inviting opportunities to reduce dementia occurrence in an increasingly aging population. However, dementia-associated metabolic risk factors are diverse, rarely occur on their own, and have complex interrelationships, making it difficult to establish clear pathological pathways. Understanding the relationships between metabolic profiles and dementia, and the key drivers of these associations, may help inform on disease pathways and approaches to prevention.

Using a data-driven self-organising map (SOM) machine-learning approach, our group previously established six metabolic subgroups, based on the clustering patterns of a panel of over 30 clinical serum, urinary, and physiological biomarkers (relating to cardiovascular health, kidney and liver function, diabetes, bone and joint, cancer and growth, and adiposity; see Supplementary text) [10]. These diverse subgroups have shown notable differences in risk of a range of age-related non-communicable diseases [10, 11], demonstrating the utility of these subgroups in comparative disease risk analyses. Lean Subgroup IV, which has a favourable lipid profile, is considered the most metabolically healthy, and indeed demonstrates the lowest risk of diabetes, hypertension, and overall cancer of the subgroups, whereas high adiposity Subgroups II (with liver stress) and III (with inflammation and kidney stress) have been linked to high risk of ischemic heart disease, hypertension, dementia, cancer, and mortality [10, 11]. Recently, we reported differential associations of these six SOM-defined subgroups (and their biomarker components) with six preclinical dementia-related neuroimaging measures, using large scale (*N* > 26,000) MRI data from the UK Biobank (UKB) [12]. In the current study, we now investigate their relationships with the incidence of ‘all-cause’ dementia and the most common dementia subtypes, Alzheimer’s disease (AD) and vascular dementia (VaD), among a population of over 300,000 white British participants.

## Methods

### Study population

The UKB is an ongoing prospective cohort study with over 500,000 participants recruited between March 2006 and July 2010. Participants were aged between 37–73 years (99.5% were 40—69 years) at recruitment which was done across 22 centres in England, Scotland, and Wales [13]. Data collection at baseline involved self-reported touchscreen questionnaires, interviews, physical measurements, and the provision of blood and urine samples for various assays. Health and medical records were linked, to obtain participants' health status [13]. For our current analysis, we focused on unrelated white British individuals with complete metabolic subgroup information (Supplementary Fig. 1). We excluded individuals with stroke (*n* = 5219) or dementia (*n* = 141) diagnoses at baseline and those without covariate information (*n* = 16,529). This resulted in an analysis of 308,019 participants, with 5,445 incident dementia cases, 2,617 incident AD cases, and 1,212 incident VaD cases (Supplementary Fig. 1). All participants provided informed consent before data collection by the UKB. The study was approved by the National Information Governance Board for Health and Social Care and North West Multicentre Research Ethics Committee (11/NW/0382). The current study was conducted under project number 89630.

### Dementia diagnosis

Case ascertainment relied on the International Classification of Diseases (ICD-10) codes, utilising first occurrences (category 1712) and algorithmically defined outcomes (category 42) from UKB resources (Supplementary Table 1). Both resources incorporated hospital inpatient data, death registry records, and self-reported medical information. The first occurrences dataset also encompassed primary care data to define health outcomes, achieving a concordance rate of over 93% for dementia and its subtypes based on the two resources. All-cause dementia was defined using ICD-10 codes F00, F01, F02, F03, and G30, while AD and VaD were specifically defined using F00 and F01 codes, respectively. Cases recorded after the baseline assessment were categorised as incident cases. The time at risk for dementia was considered from baseline until diagnosis, death, or the most recent update of hospital admission data (December 13, 2022), whichever occurred first.

### Metabolic subgroups and biomarkers

As previously described [10], study participants were divided into six metabolic subgroups based on biochemical and physiologic traits using a SOM approach, an artificial neural network technique designed to detect multivariable patterns in complex datasets. Traits used to sort individuals into subgroups included biomarkers related to cardiovascular health, diabetes, inflammation, kidney function, liver function, bone and joint health, and cancer, as well as physiological measures (Supplementary text). Each subgroup was assigned a descriptive name based on its most characteristic or defining traits; Subgroup I (“High apolipoprotein B and blood pressure without hyperglycaemia”), Subgroup II (“High triglycerides and liver enzymes”), Subgroup III (“High body mass index (BMI), C-reactive protein (CRP), and cystatin C”), Subgroup IV (“High high-density lipoprotein cholesterol (HDLC) and low BMI”), Subgroup V (“High sex hormones (oestradiol, and testosterone in males) and low calcium”) and Subgroup VI (“High urinary excretion without kidney stress”). These names are intended for descriptive purposes, and the defining traits were not necessarily those driving association with dementia risk. For extended information on the biomarker profiles of the six subgroups, please refer to Supplementary Fig. 2, or to Mulugeta et al. (2022) [10] where the subgroups were first defined. We analysed dementia risk associations for all individual biomarkers that were used to construct the metabolic subgroups. We additionally included basal metabolic rate (BMR) in these analyses due to its potential relevance in relation to dementia [12, 14], and as the related impedance measures were also components of the SOM subgrouping. We log-transformed the biomarkers to approximate a normal distribution and then standardised them to have a mean of zero and a standard deviation of one, separately for men and women.

### Covariates

Covariate information was self-reported at baseline, with the exception of the Townsend Deprivation Index, which relied on participants' postal codes as recorded in the National Health Service primary care trust registries [15]. The basic covariates encompassed age, sex, and assessment centre. Socioeconomic variables included education (categorised as none, National Vocational Qualification (NVQ)/Certificate of Secondary Education (CSE)/Advanced Levels (A-levels), or degree/professional), employment status (classified as none, retired, 1st quartile working hours (lower working hours), 2nd quartile working hours, 3rd quartile working hours, or 4th quartile working hours (higher working hours)), and Townsend Deprivation Index (differentiating between low and high deprivation based on values below and above the median, respectively). We also considered lifestyle factors, such as smoking (categorised as “never”, “ex-smoker”, or “current smoker”), alcohol consumption (categorised as “daily or almost daily”, “3 or 4 times a week”, “1 or 2 times a week”, “1 to 3 times a month”, “special occasions only”, or “never”), physical activity (categorised as “none”, “light/moderate”, or “strenuous activity”), stress-related events in the last two years (yes or no), including types of events (“serious illness, injury, or assault to yourself”, “death of a spouse or partner”, or “financial difficulties”), and adherence to a healthy diet. A ‘healthy diet’ was defined using a score (value between 0 to 4) adapted from the American Heart Association (AHA) guidelines. It included three components: 1) daily intake of > 4.5 servings of fruits and vegetables (coded as 1, otherwise 0); 2) consumption of fish at least twice a week (coded as 1, otherwise 0); and 3) limited meat intake to two or fewer servings of processed meat and five or fewer servings of red meat per week (coded as 1, otherwise 0). For analysis involving systolic and diastolic blood pressure, we accounted for changes in blood pressure due to antihypertensive medication by adding 15 mm Hg for systolic and 10 mm Hg for diastolic blood pressure for individuals who reported taking antihypertensives at the baseline (19.9%) [16]. There are three main *APOE* alleles based on the combination of variants at single nucleotide polymorphisms (SNPs) rs429358 and rs7412, with the *APOE*-ε4 allele having the haplotype rs429358-C, rs7412-C. Individuals were categorised as a ‘homozygous ε4 carrier’ if they possessed ‘C/C’ variants for both SNPs, as ‘heterozygous ε4 carrier’ if they possessed ‘C/T’ for rs429358 and ‘C/T’ or ‘C/C’ for rs7412, or if they possessed ‘C/C’ for rs429358 and ‘C/T’ for rs7412. Any other allele combinations for the two variants were considered to be ε4 noncarriers.

### Statistical analysis

We calculated the incidence rate per 10,000 person-years at risk for all-cause dementia, AD, and VaD across different baseline characteristics of the participants. Cox proportional hazard regression was employed to estimate the hazard ratio (HR) and the 95% confidence interval (95% CI) for the relationship between various metabolic subgroups and the dementia outcomes. Subgroup IV was used as the referent group, reflecting a ‘metabolically favourable’ profile characterised by high HDLC and low BMI [10], low volume of white matter hyperintensities (WMH; a marker of white matter lesions), and the greatest hippocampal volume of all the subgroups [12]. Our analysis included four adjustment models: first, adjusted for basic covariates; second, adjusted for both basic and socioeconomic covariates; third, adjusted for basic and lifestyle covariates; and the fourth, adjusted for all covariates above. For subgroup-dementia analyses, *p*-values < 0.05 were considered significant.

Since the ε4 allele of the *APOE* gene, a strong genetic risk factor for dementia, is associated with many metabolic biomarker traits in the UKB population (e.g., lower CRP and BMI, unfavourable lipid profile, lower glycated haemoglobin (HbA1c)) [17] in sensitivity analyses we additionally included in the fully adjusted model, adjustment for the number of *APOE-*ε4 alleles carried. We also tested whether *APOE*-ε4 allele count (0, 1, or 2) modified the subgroup–dementia associations using likelihood ratio tests through comparing the model before and after including the *APOE*-ε4–subgroup interaction term, and conducted stratified analyses when interactions were detected.

The proportional hazards assumption was verified using Schoenfeld residuals (*p* > 0.19). In the linear biomarker analyses, we conducted additional tests for three-way interactions of biomarkers with age and sex, two-way interactions with age, and with sex, and two-way interactions with number of *APOE*-ε4 alleles, to investigate effect modification. In the presence of interaction, stratified analysis was performed. To further understand the dose–response association between the biomarkers and the outcomes, we employed restricted cubic spline regression within Cox proportional hazard models, adjusting for basic, socioeconomic, and lifestyle covariates. We compared Akaike Information Criterion (AIC) in the models (for all dementia outcomes) using three, four, or five knots, which suggested the best performance for the model with three knots (Supplementary Table 2). These knots were placed at the 10th, 50th, and 90th percentiles of the biomarker distributions as recommended by Harrell [18]. The IGF-1–dementia analysis was the only one that showed better performance with four knots, and for this biomarker we repeated the analysis using four knots placed at the 5th, 35th, 65th, and 95th percentiles of the IGF-1 distribution [18]. Evidence of nonlinearity was determined using a likelihood ratio test comparing the linear model with the spline model. For nonlinear associations, we also assessed two-way interactions with *APOE*-ε4 allele count; no such interactions were detected.

Given the multiple tests conducted for 39 biochemical and physiologic traits, we applied a Bonferroni-corrected significance threshold (*p* < 0.0013) for statistical decisions regarding biomarker analyses. For sex hormone related biomarkers (oestradiol, testosterone and SHBG), stratification by sex was undertaken if the sex interaction *p*-value was < 0.05. All observational analyses were conducted using STATA SE version 17.0 and R 4.3.2 software.

## Results

### Population characteristics

We included 308,019 white British participants, with 53.5% being female and 44.7% aged over 60 years (Table 1**,** and extended information in Supplementary Table 3). The incidence rate of all-cause dementia was 13.2 (*n* = 5445) per 10,000 person-years at risk. The median age at diagnosis was 71.4 years (interquartile range: 64.1 to 76.5 years). For AD and VaD, the incidence rates were 6.3 and 2.9 per 10,000 person-years at risk (2617 and 1212 cases), respectively. There were 3336 participants with other types of dementia (752 (23%) frontotemporal, and 2584 (77%) of unspecified type). Incidence rates for dementia and its subtypes were higher among males, the oldest age group (60–72 years at baseline), retirees, those unemployed, individuals with no educational qualifications, those living in highly deprived areas, and non-consumers of alcohol (for all, *p* ≤ 0.03). Conversely, non-smokers and physically active individuals had a lower incidence of dementia, AD, and VaD (for all, *p* ≤ 0.003).

**Table 1:** Incidence rates for dementia, Alzheimer’s disease and vascular dementia by characteristics of the UK Biobank participants

		*N* (%)	Person-years at risk for dementia	Incidence rate* (*n*) for dementia^†^	Incidence rate* (*n*) for Alzheimer’s disease^†^	Incidence rate* (*n*) for vascular dementia^†^
Total		308,019	4,126,811	13.2 (5445)	6.3 (2617)	2.9 (1212)
Sex						
Male		143,351 (46.5)	1,899,769	15.2 (2891)	6.7 (1275)	3.8 (725)
Female		164,668 (53.5)	2,227,042	11.5 (2554)	6.0 (1342)	2.2 (487)
	*p*			2.7 × 10^-22^	0.03	6.7 × 10^-20^
Age at baseline						
39 – 49 years		68,384 (22.2)	943,238	0.8 (74)	0.2 (22)	0.1 (9)
50 - 59 years		102,083 (33.1)	1,388,323	4.2 (585)	1.6 (228)	0.8 (110)
60 - 72 years		137,552 (44.7)	1,795,251	26.7 (4786)	13.1 (2367)	6.1(1093)
	*p*			< 1.0 × 10^-300^	9.7 × 10^-262^	8.8 × 10^-117^
Education						
None		51,268 (16.6)	670,282	27.8 (1865)	13.8 (927)	6.8 (461)
NVQ/CSE/A-levels		110,268 (35.8)	1,479,898	11.4 (1681)	5.6 (827)	2.6 (379)
Degree/professional		146,483 (47.6)	1,976,631	9.6 (1899)	4.4 (863)	1.9 (372)
	*p*			1.0 × 10^-64^	3.5 × 10^-35^	7.0 ×10^-26^
Employment						
No		23,810 (7.7)	313,489	11.7 (368)	4.6 (144)	3.3 (104)
Retired		107,655 (38.9)	1,404,535	28.5 (3998)	14.1 (1988)	6.2 (881)
1^st^ quartile (Lower working hours)		43,278 (14.1)	586,864	6.7 (395)	3.0 (178)	1.6 (93)
2^nd^ quartile working hours		29,886 (9.7)	408,311	4.7 (192)	2.0 (83)	0.9 (38)
3^rd^ quartile working hours		55,805 (18.1)	763,050	3.7 (285)	1.8 (139)	0.8 (60)
4^th^ quartile (Higher working hours)		47,585 (15.5)	650,563	3.2 (207)	1.3 (85)	0.6 (36)
	*p*			9.6 × 10^-113^	9.9 × 10^-46^	4.9 × 10^-28^
Townsend deprivation index						
Less deprived (below median)		153,958 (50.0)	2,076,406	12.2 (2535)	5.9 (1237)	2.6 (541)
Highly deprived (above median)		154,061 (50.0)	2,050,406	14.2 (2910)	6.7 (1380)	3.2 (671)
	*p*			3.5 × 10^-18^	3.1 × 10^-07^	9.1 × 10^-09^
Smoking						
Never		169,490 (55.0)	2,294,265	11.0 (2515)	5.4 (1238)	2.3 (519)
Ex-smoker		108,128 (35.1)	1,436,014	16.6 (2389)	7.9 (1142)	3.7 (538)
Current		30,401 (9.9)	396,532	13.6 (541)	5.9 (237)	3.9 (155)
	*p*			6.2 × 10^-10^	0.003	5.1 × 10^-08^
Alcohol						
Daily or almost daily		66,688 (21.7)	889,462	13.3 (1183)	5.9 (523)	3.0 (268)
3 or 4 times a week		75,455 (24.5)	1,018,060	10.6 (1082)	5.3 (540)	2.3 (235)
1 or 2 times a week		81,176 (26.3)	1,092,805	11.7 (1283)	5.9 (648)	2.5 (279)
1 to 3 times a month		34,028 (11.0)	457,695	11.8 (538)	5.7 (261)	2.6 (119)
Special occasions only		31,389 (10.2)	416,460	17.0 (708)	8.5 (353)	3.8 (160)
Never		19,283 (6.3)	252,329	25.8 (651)	11.5 (292)	6.0 (151)
	*p*			8.8 × 10^-61^	6.7 × 10^-20^	7.7 × 10^-17^
Physical activity						
None		17,079 (5.6)	221,965	21.0 (467)	8.7 (193)	5.1 (114)
Light/moderate		258,249 (83.8)	3,459,231	13.67 (4724)	6.6 (2292)	3.0 (1049)
Strenuous activity		32,691 (10.6)	445,615	5.7 (254)	3.0 (132)	1.1 (49)
	*p*			6.6 × 10^-34^	2.0 × 10^-06^	6.9 × 10^-12^
Serious illness, injury, or assault to yourself in the last 2 years						
No		281,179 (91.3)	3,783,693	12.6 (4757)	6.2 (2347)	2.7 (1035)
Yes		26,840 (8.3)	343,118	20.1 (688)	7.8 (270)	5.1 (177)
	*p*			1.2 × 10^-21^	0.009	6.1 × 10^-11^
Death of a spouse or partner in last 2 years						
No		303,264 (98.5)	4,064,261	13.0 (5300)	6.3 (2546)	2.9 (1185)
Yes		4,755 (1.5)	62,551	23.2 (145)	11.3 (71)	4.2 (27)
	*p*			6.7 × 10^-4^	0.03	0.54
Healthy diet score						
0 (least healthy)		1,389 (0.5)	18,126	17.1 (31)	4.9 (9)	3.8 (7)
1		43,061 (14.0)	573,364	11.8 (674)	5.1 (292)	2.6 (152)
2		115,022 (37.3)	1,540,803	12.5 (1922)	6.1 (939)	2.8 (437)
3		105,447 (34.2)	1,414,394	13.9 (1963)	6.9 (975)	3.0 (422)
4 (most healthy)		43,100 (14.0)	580,124	14.7 (855)	6.9 (402)	3.3 (194)
	*p*			0.18	0.65	0.59

*Incidence rates are per 10,000 person-years at risk

† *p*-value is from a likelihood ratio test in a logistic regression model adjusting for age, sex, and assessment centre

A-level Advanced level; CSE Certificate of Secondary Education; NVQ National Vocational Qualification

### Metabolic subgroups and incidence of dementia

In the basic covariate adjustment model, controlling for age, sex, and assessment centre, the two metabolic subgroups with greatest adiposity showed the highest incidence of dementia in comparison to referent Subgroup IV (with high HDLC and low BMI); Subgroup II with liver stress (HR 1.23, 95% CI 1.12 to 1.35) and Subgroup III with inflammation and kidney stress (1.28, 1.17 to 1.40). These associations were attenuated by accounting further for lifestyle factors (Table 2, Supplementary Table 4 A).

**Table 2:** Metabolic subgroup associations with dementia

	All-cause dementia				Alzheimer’s disease				Vascular dementia			
	HR				HR				HR			
	95%CI				95%CI				95%CI			
Adjustment model:	B	B+S	B+L	B+S+L	B	B+S	B+L	B+S+L	B	B+S	B+L	B+S+L
I. High ApoB and BP without hyperglycaemia	0.97	0.93	0.93	0.93	0.92	0.89	0.90	**0.90**	1.08	1.04	1.05	1.05
	0.89–1.05	0.86–1.02	0.85–1.02	0.84–1.00	0.82–1.04	0.79–1.00	0.79–1.01	**0.78**–**0.99**	0.89–1.31	0.86–1.26	1.87–1.28	0.85–1.25
II. High triglycerides and liver enzymes	**1.23**	**1.13**	1.09	1.04	1.08	1.00	0.98	0.94	**1.83**	**1.64**	**1.62**	**1.52**
	**1.12–1.35**	**1.03–1.25**	0.99–1.20	0.95–1.15	0.94–1.24	0.87–1.15	0.85–1.12	0.82–1.08	**1.51–2.21**	**1.35–1.98**	**1.33–1.97**	**1.25–1.85**
III. High BMI and CRP and cystatin C	**1.28**	**1.18**	1.09	1.05	1.00	0.93	**0.87**	**0.85**	**1.97**	**1.76**	**1.66**	**1.58**
	**1.17–1.40**	**1.08–1.29**	0.99–1.19	0.96–1.15	0.88–1.14	0.81–1.06	**0.76–1.00**	**0.74–0.97**	**1.65–2.36**	**1.46–2.11**	**1.38–2.00**	**1.31–1.90**
IV. High HDLC and low BMI (Referent)	1.00	1.00	1.00	1.00	1.00	1.00	1.00	1.00	1.00	1.00	1.00	1.00
V. High sex hormones and low calcium	1.06	1.06	1.01	1.02	0.98	0.98	0.94	0.95	1.01	1.00	0.96	0.97
	0.98–1.15	0.97–1.15	0.94–1.10	0.94–1.11	0.87–1.09	0.87–1.10	0.84–1.06	0.85–1.07	0.83–1.21	0.83–1.21	0.80–1.16	0.80–1.17
VI. High urinary excretion without kidney stress	1.00	0.99	0.95	0.95	**0.85**	**0.83**	**0.80**	**0.80**	1.08	1.06	1.03	1.03
	0.91–1.10	0.90–1.08	0.87–1.04	0.87–1.05	**0.74–0.97**	**0.72–0.95**	**0.70–0.92**	**0.70–0.92**	0.88–1.33	0.86–1.30	0.84–1.27	0.83–1.27

Cox proportional hazard regression was used to estimate the hazard ratios (HR) for risk of the dementia outcomes across the metabolic subgroups, in comparison to Subgroup IV. Findings from analyses adjusted for basic covariates (age, sex, and assessment centre), additionally adjusted for socioeconomic (B+S; education, employment status, and Townsend Deprivation Index) or lifestyle covariates (B+L; smoking, alcohol consumption, physical activity, stress-related events in the last two years (yes or no), types of stress events (“serious illness, injury, or assault to yourself”, “death of a spouse or partner”, or “financial difficulties”), and healthy diet), and fully adjusted for basic, socioeconomic, and lifestyle factors (B+S+L) are presented. Associations with *p*-values < 0.05 are shown in bold. Further *p*-value information can be found in Supplementary Table 4A

ApoB apolipoprotein B; BMI body mass index; BP blood pressure; CRP C-reactive protein; HDLC high density lipoprotein cholesterol

Subgroup VI (with high urinary excretion but not kidney stress) showed lower incidence of AD than referent Subgroup IV in the basic covariate adjusted model (0.85, 0.74 to 0.97), an association strengthened by subsequent adjustments for socioeconomic factors (0.83, 95% CI 0.72 to 0.95), lifestyle factors (0.80, 0.70 to 0.92) (Supplementary Table 4 A), and when considering all covariates (Table 2). Furthermore, Subgroup III with high BMI, kidney stress, and inflammation, showed lower risk of AD in comparison to Subgroup IV, apparent only after adjustment for lifestyle factors (0.87, 0.76 to 0.999) and in the fully adjusted model (0.85, 0.74 to 0.97), but not with basic adjustment alone (1.00, 0.88 to 1.14) (Table 2, Supplementary Table 4 A). Subgroup I (high ApoB and blood pressure without hyperglycaemia) also showed lower risk of AD in the fully adjusted model (0.90, 0.78 to 0.99). No subgroups showed a greater risk of AD than referent Subgroup IV, with Subgroups V (high sex hormones) and II (liver stress) having levels of risk “on par” with the referent group. For alternate visualisation purposes we also present the subgroup–AD association data using Subgroup VI (with the lowest AD risk) as the referent group, showing higher risk of AD in Subgroups II, IV and V, in the basic adjustment model, that persisted after full adjustment for lifestyle and socioeconomic factors (Supplementary Table 4B).

For VaD, higher incidence was observed among the two subgroups with greatest adiposity; Subgroup II with liver stress (1.83, 1.51 to 2.21) and Subgroup III with inflammation and kidney stress (1.97, 1.65 to 2.36) compared to Subgroup IV. These associations remained, although partially attenuated, after adjusting for all covariates (Table 2, Supplementary Table 4 A).

**Fig. 1:**
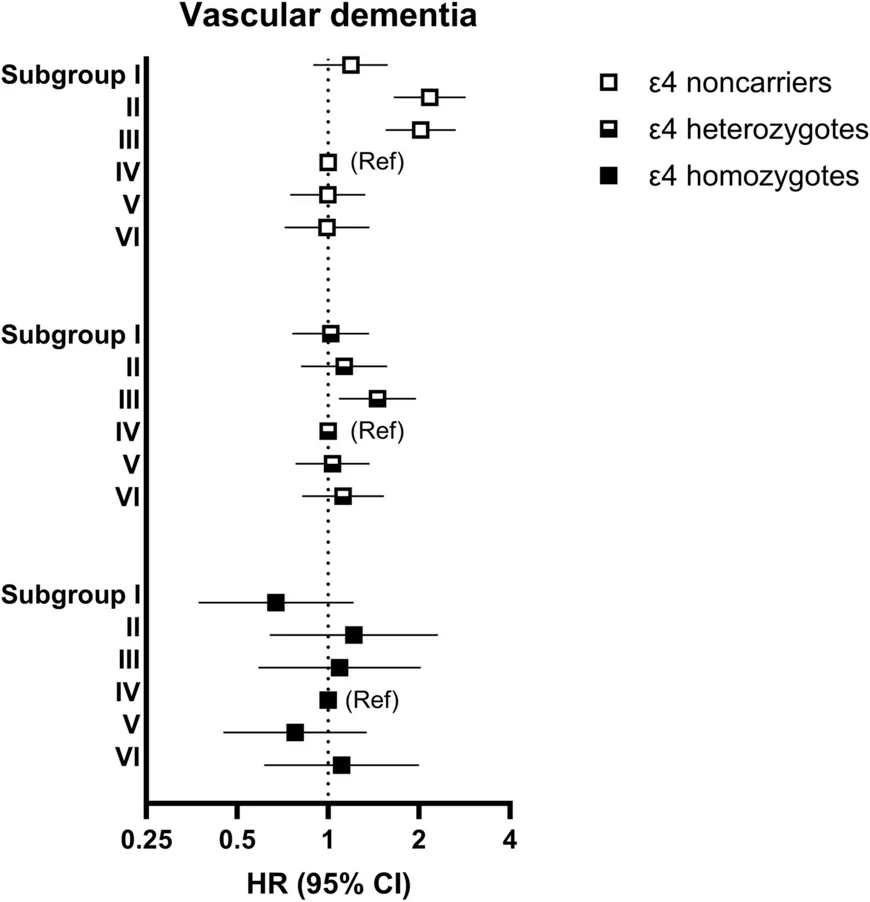
Vascular dementia risk across metabolic subgroups by number of *APOE*-ε4 alleles carried. Estimates are from Cox proportional hazard regression, adjusted for basic (age, sex, and assessment centre), socioeconomic (education, employment status, and Townsend Deprivation Index), and lifestyle (smoking, alcohol consumption, physical activity, stress-related events in the last two years (yes or no), types of stress events (“serious illness, injury, or assault to yourself”, “death of a spouse or partner”, or “financial difficulties”) and healthy diet) covariates. 95% CI, 95% confidence interval; HR hazard ratio.

In sensitivity analyses, additional adjustment for the number *APOE*-ε4 alleles made no difference to the associations with dementia outcomes in the metabolic subgroup analysis (Supplementary Table 4 C). There was evidence of an *APOE*-ε4 interaction with subgroup associations with VaD (*p*_int_ = 7.8 × 10^–4^), that was also reflected for dementia overall (*p*_int_ = 0.0016), but was not evident for AD (*p*_int_ = 0.29) (Supplementary Table 4 C). After subsequently stratifying the (fully covariate-adjusted) analysis by *APOE*-ε4 allele number (Supplementary Table 4D) we observed stronger associations of Subgroup II and Subgroup III with VaD compared to referent Subgroup IV, among noncarriers of the *APOE*-ε4 allele (HR 2.17, 95% CI 1.65 to 2.86, and 2.03, 1.55 to 2.65, respectively; Fig. 1). Subgroup III (high BMI, kidney stress, inflammation) also showed higher risk than lean Subgroup IV in ε4 heterozygotes (1.46, 1.09 to 1.95) (Fig. 1).

### Biomarkers and incidence of dementia

**Fig. 2:**
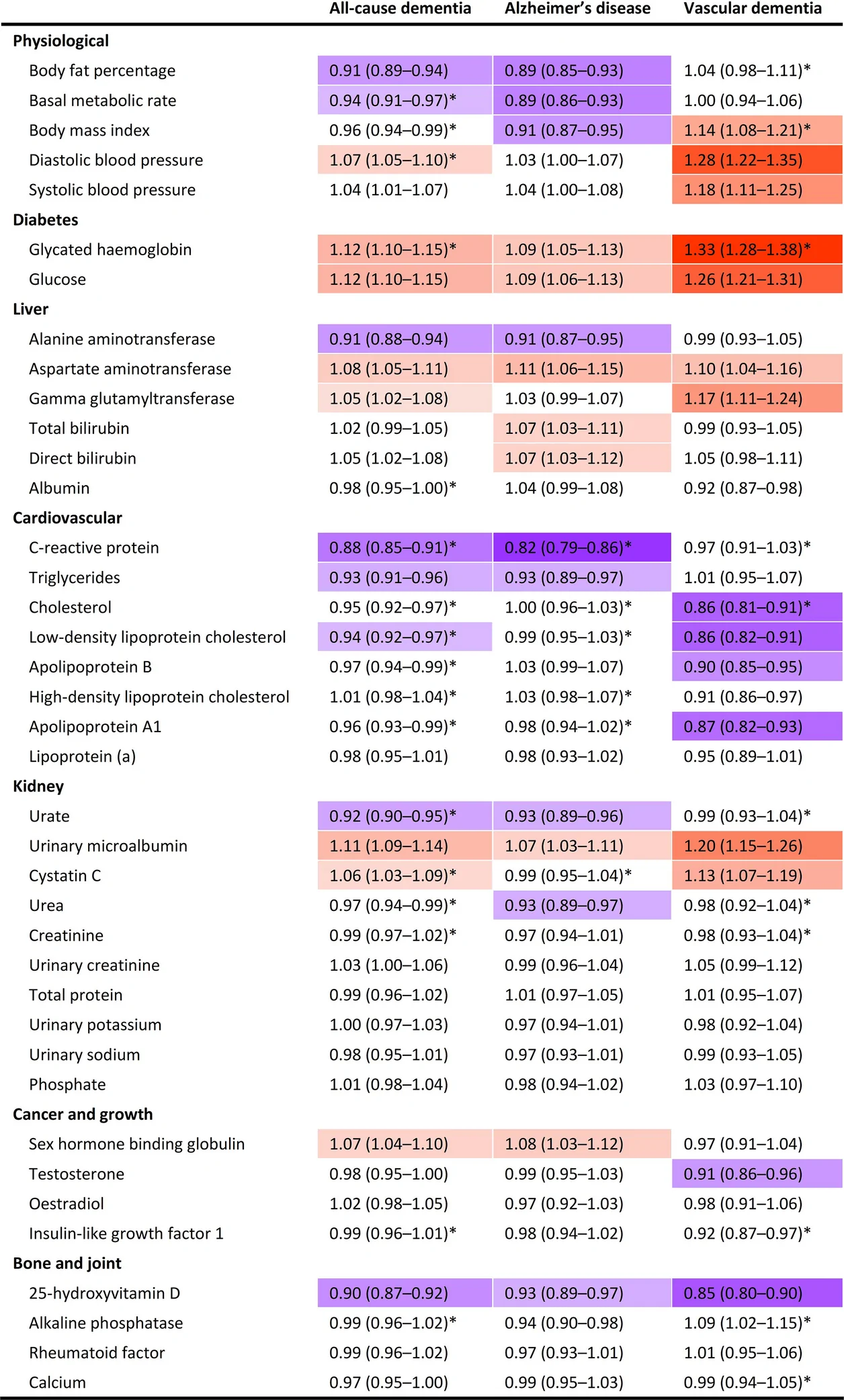
Metabolic biomarker associations with dementia. Linear relationships between biomarkers and dementia outcomes using Cox proportional hazard regression, adjusting for basic (age, sex, and assessment centre), socioeconomic (education, employment status, and Townsend Deprivation Index), and lifestyle (smoking, alcohol consumption, physical activity, stress-related events in the last two years (yes or no), types of stress events (“serious illness, injury, or assault to yourself”, “death of a spouse or partner”, or “financial difficulties”), and healthy diet) covariates. Systolic and diastolic blood pressure measures were additionally adjusted for use of blood pressure-lowering medications. Hazard ratios and 95% CI are shown, and linear associations with *p*-values < 0.0013 are indicated in colour, with orange indicating higher values, and purple indicating lower values, and more intense colour representing greater deviation of the HR point estimate from 1.00. Extended information can be found in Supplementary Table 5. * Evidence of nonlinearity being a better fit than linear model (*p*_LHR_ < 0.0013).

**Fig. 3:**
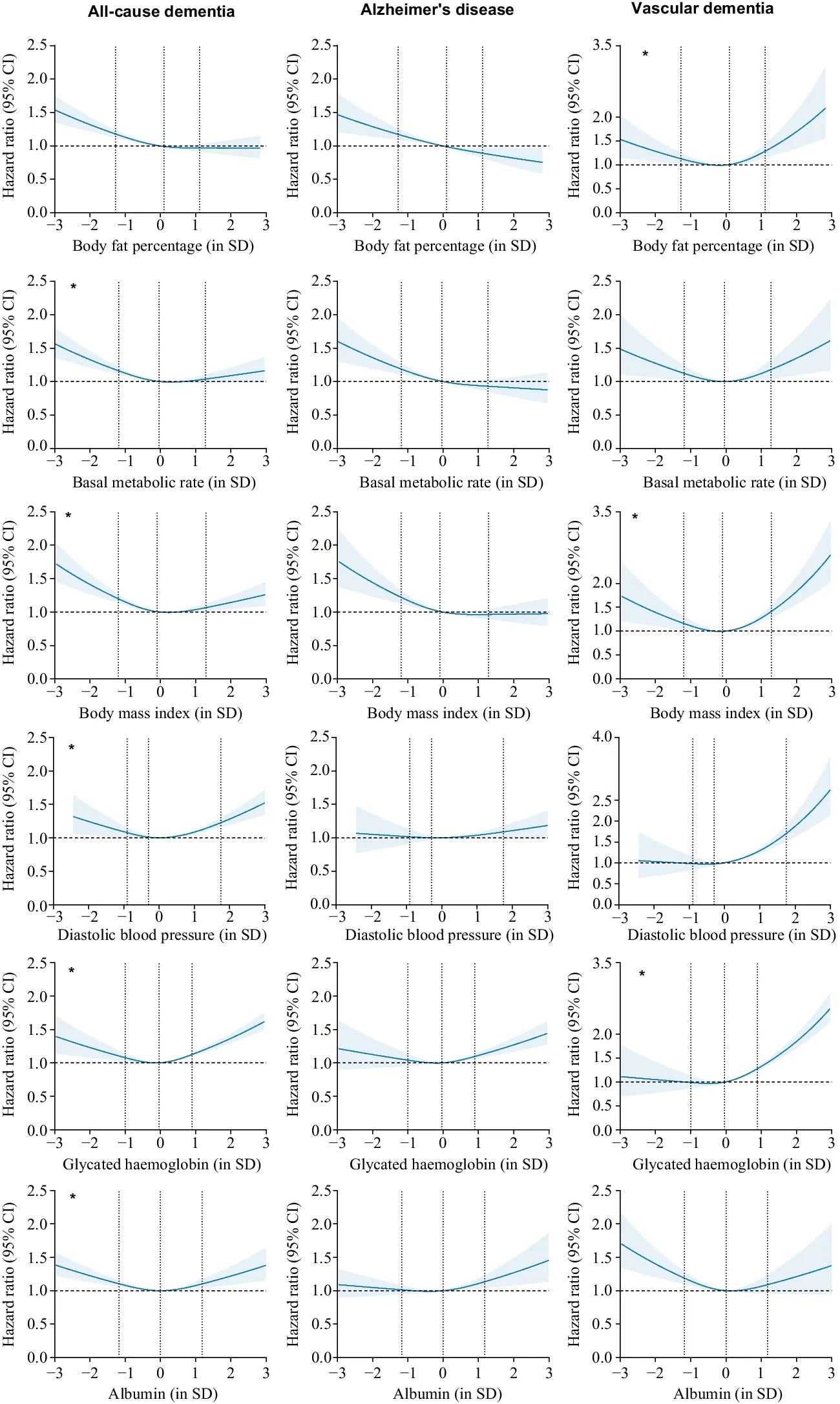

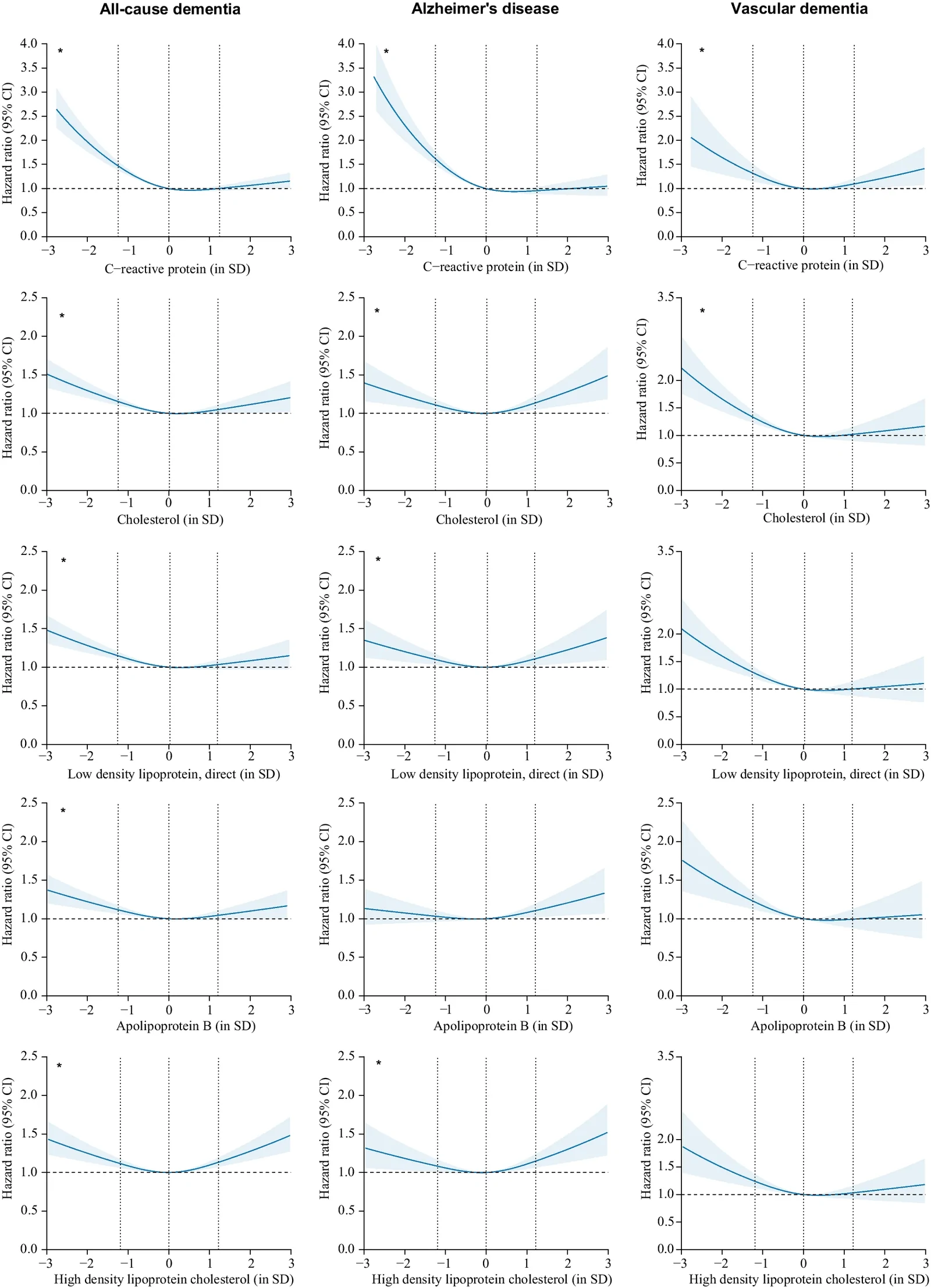

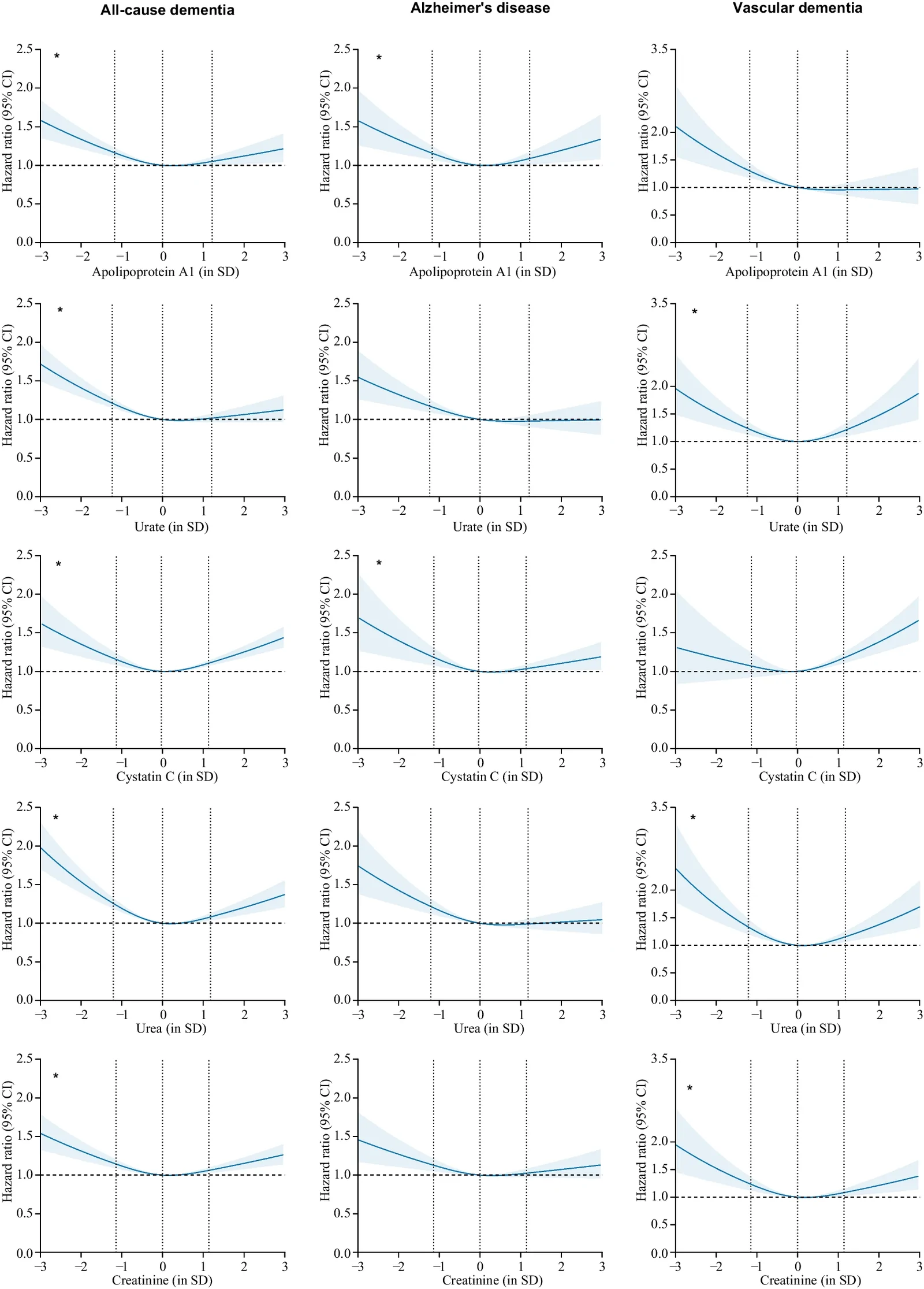

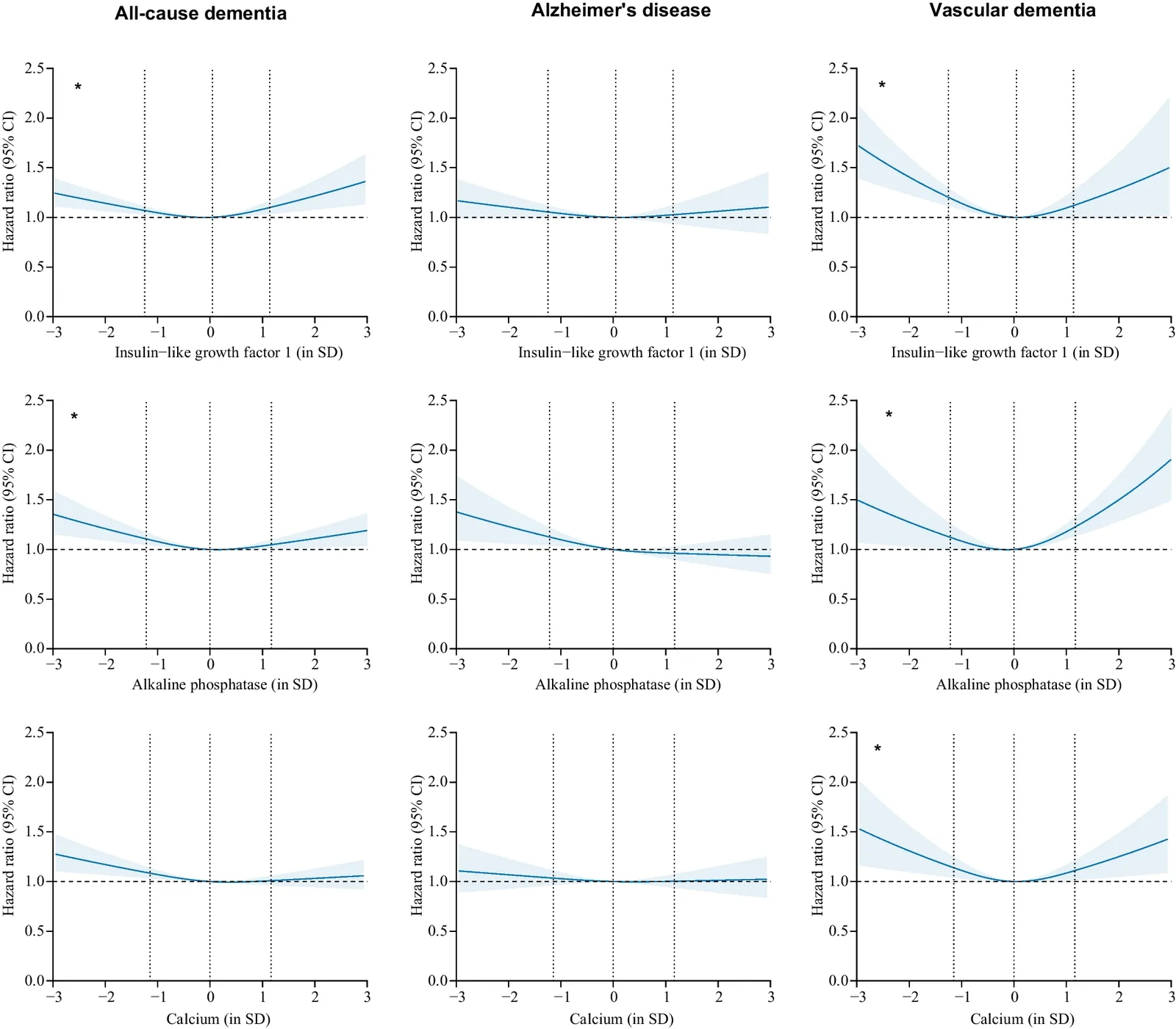
Nonlinear associations between biomarkers and risk of dementia. Restricted cubic spline models fitted for Cox proportional hazard models are shown for all-cause dementia, AD, and VaD, for biomarkers where there was evidence at the Bonferroni-adjusted threshold (*p*_LHR_ < 0.0013) of a nonlinear relationship for at least one of the dementia outcomes, as indicated with an asterisk (*). Analyses are adjusted for basic (age, sex, and assessment centre), socioeconomic (education, employment status, and Townsend Deprivation Index), and lifestyle (smoking, alcohol consumption, physical activity, stress-related events in the last two years (yes or no), types of stress events (“serious illness, injury, or assault to yourself”, “death of a spouse or partner”, or “financial difficulties”), and healthy diet) covariates. Extended data in Supplementary Fig. 3 shows spline curves for all biomarkers and dementia outcomes. 95% CI, 95% confidence interval; SD, standard deviation.

We next explored the associations between 39 biochemical and physiological traits that comprise components of the SOM subgroups, and the dementia outcomes, to inform on which biomarkers might be driving the subgroup associations. For all outcomes, a summary of linear associations, indicating those that showed evidence of superior fit with nonlinear modelling, is shown in Fig. 2. Spline curves for biomarkers showing evidence of nonlinearity (passing Bonferroni threshold *p* < 0.0013) for at least one dementia outcome are shown in Fig. 3, with Supplementary Fig. 3 showing spline curves for all biomarkers. Extended data for biomarker analyses is provided in Supplementary Table 5.

#### Biomarkers and incidence of Alzheimer’s disease

Several measures related to body composition were associated with risk of AD. Body fat percentage (HR per 1 SD higher log-transformed measure 0.89, 95% CI 0.85 to 0.93), BMI (0.91, 0.87 to 0.95), and BMR (0.89, 0.86 to 0.93) were inverse-linearly associated with risk (Fig. 2). Among cardiovascular biomarkers, having lower triglycerides was linearly associated with higher risk (Fig. 2), while nonlinear relationships were seen for several cholesterol-related markers (including total cholesterol, apolipoprotein A1 (ApoA1), HDLC, and low-density lipoprotein cholesterol (LDLC)); spline curves suggesting U-shaped relationships with AD risk for these markers (Fig. 3). Systemic inflammation marker CRP showed a nonlinear (L-shaped) relationship with AD (Fig. 3). Measures of higher blood sugar were linearly associated with greater risk (HbA1c HR 1.09, 1.05 to 1.13; similar for glucose), while 25-hydroxyvitamin D (25(OH)D) showed an inverse linear association (0.93, 0.89–0.97; Fig. 2). Among liver-related biomarkers, higher aspartate aminotransferase (AST) and lower alanine aminotransferase (ALT) were linearly associated with higher AD risk, as were higher bilirubin levels (direct and total measures) (Fig. 2). Among kidney-related biomarkers, microalbumin showed a positive linear association with AD risk, urate and urea were inversely associated with risk, while cystatin C showed a nonlinear association, showing an inverse relationship in the below mean range of cystatin C. Among cancer and growth-related biomarkers, sex hormone binding globulin (SHBG) showed a positive linear association with AD risk. Please refer to Fig. 2 and Fig. 3 for linear and nonlinear associations, respectively, with dementia outcomes.

**Fig. 4:**
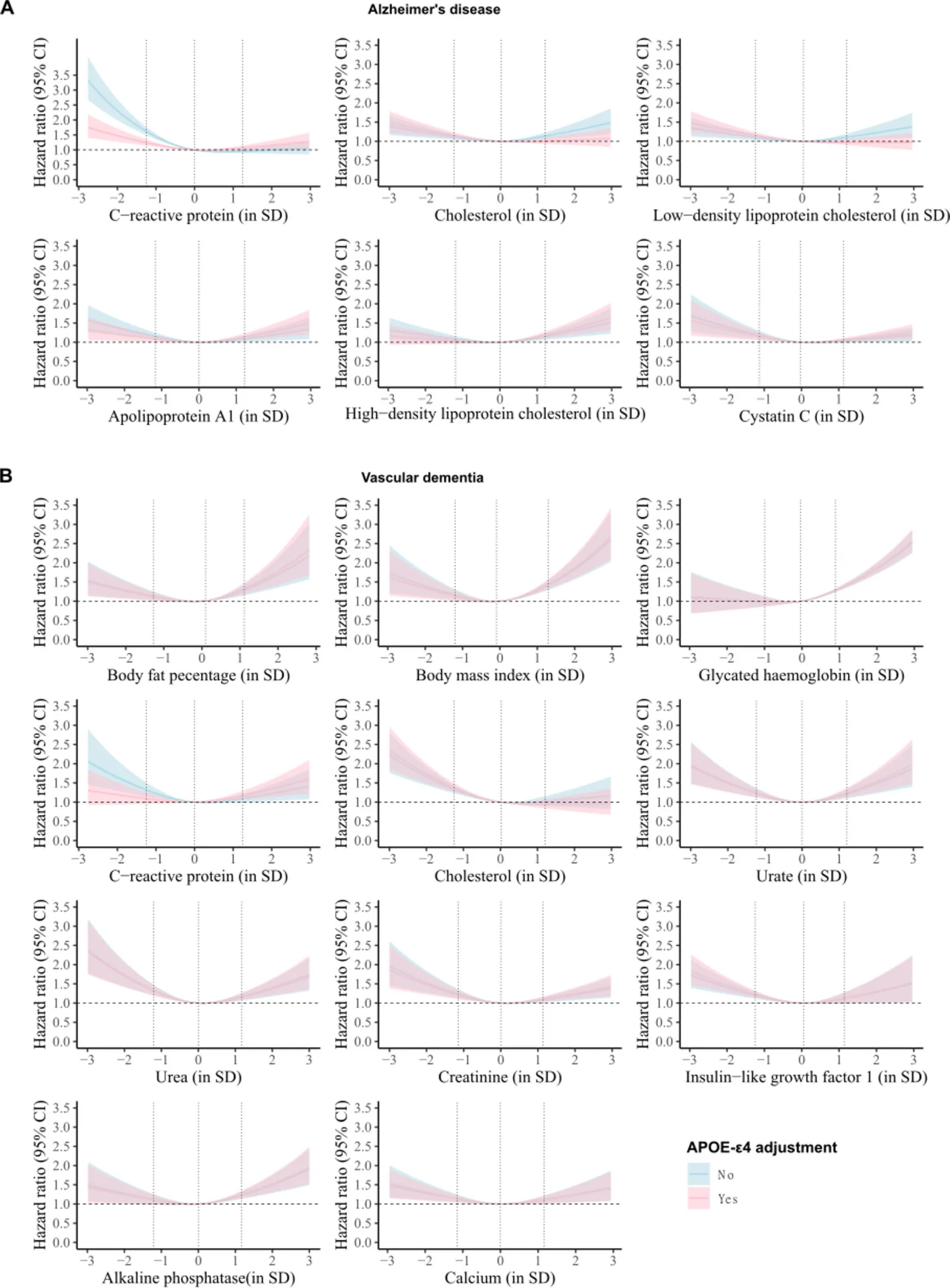
Effects of *APOE*-ε4 adjustment on nonlinear biomarker associations with Alzheimer’s disease and vascular dementia. Restricted cubic spline models fitted for Cox proportional hazard models without (blue) and with (red) adjustment for number of *APOE*-ε4 alleles are shown for biomarkers that showed nonlinear associations with **A**) Alzheimer’s disease, and **B**) vascular dementia in the main analysis. Analyses are adjusted for basic (age, sex, and assessment centre), socioeconomic (education, employment status, and Townsend Deprivation Index), and lifestyle (smoking, alcohol consumption, physical activity, stress-related events in the last two years (yes or no), types of stress events (“serious illness, injury, or assault to yourself”, “death of a spouse or partner”, or “financial difficulties”), and healthy diet) covariates. 95% CI, 95% confidence interval; SD, standard deviation.

In sensitivity analyses, no *APOE*-ε4 interactions with biomarker–AD associations were detected (Supplementary Table 5). Adjustment for *APOE*-ε4 allele number had minimal impact on linear biomarker associations with this dementia subtype, except for exposing an inverse association between ApoB and AD (HR 0.92, 95% CI 0.89 to 0.96) that was not evident in the main analysis (1.03, 0.99 to 1.07) (Supplementary Table 5, Supplementary Fig. 4). Nonlinear associations between cholesterol biomarkers and AD also appeared to be influenced by the known associations of *APOE*-ε4 with lower HDLC, and higher LDLC and cholesterol [17, 19] (Fig. 4A). *APOE*-ε4 adjustment markedly attenuated the L-shaped association between CRP and AD but did not abolish it (Fig. 4A).

#### Biomarkers and incidence of vascular dementia

Some biomarkers showed a similar association with VaD as for AD. For example, diabetes-related biomarkers were associated with more VaD risk; glucose having a linear association (HR per 1 SD higher log-transformed measure 1.26, 1.21 to 1.31; Fig. 2), and HbA1c showing a nonlinear association with VaD (Fig. 3). Furthermore, 25(OH)D showed an inverse linear association with VaD risk (0.85; 0.80–0.90), while kidney biomarker microalbumin (1.20, 1.15–1.26) and liver enzyme AST (1.10; 1.04–1.16) showed positive linear associations with VaD risk.

For other biomarkers the relationships with VaD contrasted to those for AD. Measures of adiposity and inflammation showed nonlinear relationships with risk of VaD, with generally U-shaped risk associations for BMI, body fat percentage and CRP (Fig. 3). Liver enzyme gamma glutamyltransferase (GGT) showed a notable positive linear association with VaD (1.17, 1.11 to 1.24) as did (blood pressure medication-adjusted) diastolic blood pressure (HR 1.28, 1.22 to 1.35) and systolic blood pressure (1.18; 1.11 to 1.25) (Fig. 2). Among lipid-related cardiovascular biomarkers, cholesterol showed a nonlinear (L-shaped) association with VaD. Alkaline phosphatase and calcium showed U-shaped associations with VaD risk, as did kidney biomarkers urate, urea and creatinine (Fig. 3). Testosterone was inverse-linearly associated with VaD risk (0.91, 0.86 to 0.96), while insulin-like growth factor 1 (IGF-1) showed a U-shaped association (with tighter error margins in the below-mean IGF-1 range).

**Fig. 5:**
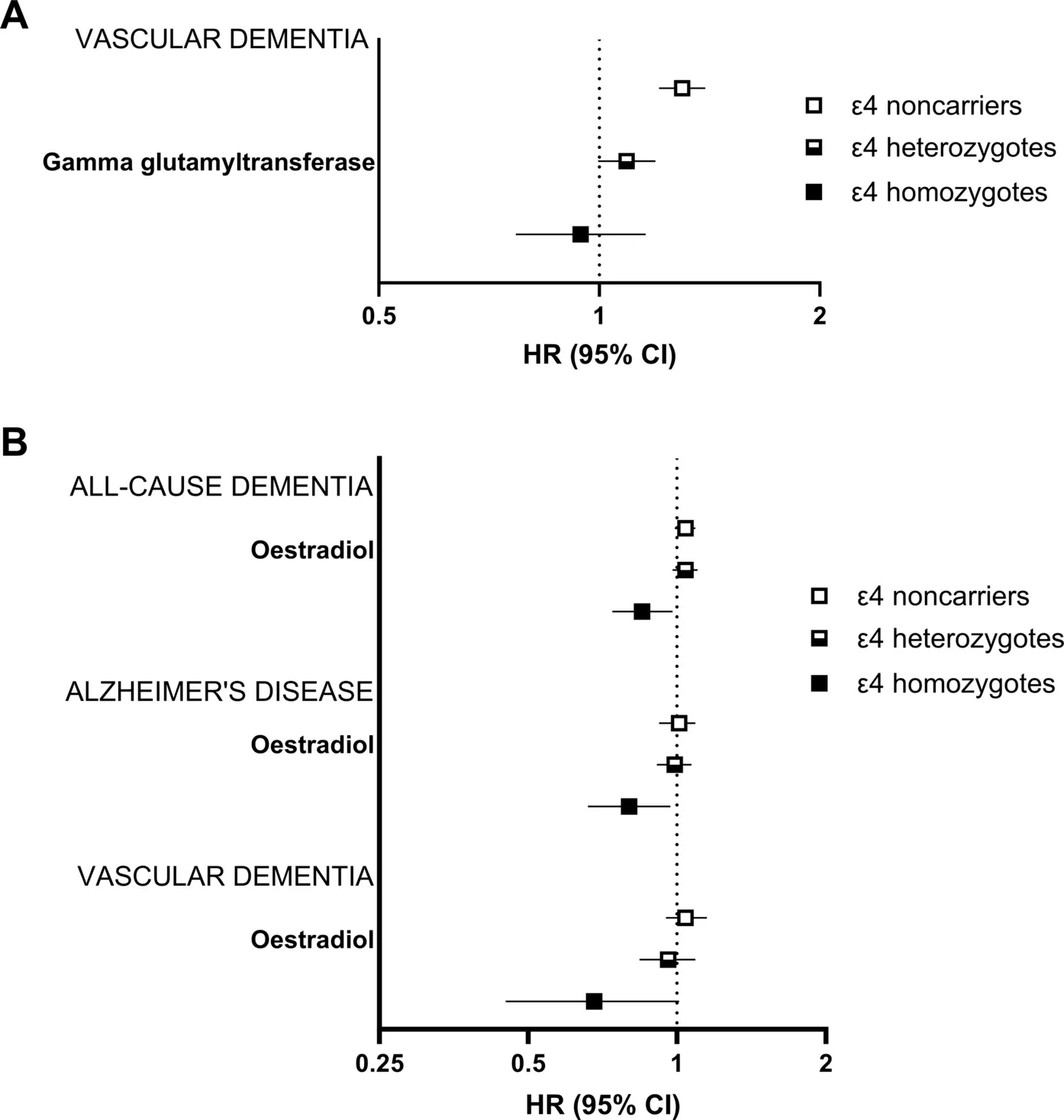
Biomarker associations with dementia outcomes, stratified by *APOE*-ε4 allele number. **A**) Association of gamma glutamyltransferase with vascular dementia by number of *APOE*-ε4 alleles shows highest risk in ε4 noncarriers. **B**) Association of oestradiol with all-cause dementia by number of *APOE*-ε4 alleles shows lower risk in those homozygous for the ε4 allele. A similar trend is observed for dementia subtypes Alzheimer’s disease and vascular dementia. 95% CI, 95% confidence interval; HR, hazard ratio.

The association of diastolic blood pressure with VaD showed an interaction by age (*p*_int_ = 1.6 × 10^–5^) and was stronger among individuals aged under 65 years (HR 1.45, 95% CI 1.34 to 1.58) than in those aged 65 and over (1.28, 1.22 to 1.35), whereas the testosterone–VaD association showed an interaction by sex (*p*_int_ = 4.07 × 10^–3^), being evident in males (0.86, 0.81 to 0.92), but not in females (1.02, 0.93–1.14) (Supplementary Table 6). The linear association of GGT with VaD differed by number of *APOE*-ε4 alleles (*p*_int_ = 4.3 × 10^–5^), with ε4 noncarriers having the greatest risk (1.30, 1.21 to 1.40; Fig. 5, Supplementary Table 5). Adjustment for *APOE*-ε4 had little-to-no impact on linear and nonlinear biomarker–VaD associations (Supplementary Table 5, Fig. 4B, Supplementary Fig. 4), most notably shifting the U-shaped association to a J-shaped association for CRP (Fig. 4B).

#### Biomarkers and incidence of all-cause dementia

For all-cause dementia, biomarker associations generally reflected a combination of the associations of the main subtypes, AD and VaD, described above (Fig. 2, Fig. 3, Supplementary Table 5). Although oestradiol was not associated with dementia in the main analysis, an *APOE*-ε4 interaction was detected with the linear model for this biomarker (*p*_int_ = 1.1 × 10^–4^), with analyses stratified by ε4 allele number showing nominally lower risk of dementia by higher oestradiol in ε4 homozygotes (Fig. 5B**)**. There was also some evidence for an interaction by age for linear (Supplementary Table 5) and nonlinear (Supplementary Fig. 5) IGF-1–dementia models. Please refer to Supplementary Table 5 for extended data from all biomarker adjustment and interaction analyses.

## Discussion

In this large population-based study we investigated relationships between SOM-derived metabolic subgroups [10] and individual biomarkers, and dementia outcomes, finding contrasting risks across the six diverse profiles. More specifically, we found that high adiposity subgroups with liver stress, or kidney stress and inflammation, showed more risk of VaD, while subgroups with hyperglycaemia or low adiposity had higher risk of AD. Biomarkers that associated with the different dementia subtypes differentially cluster across the metabolic subgroups. Our findings inform on the metabolic diversity underpinning dementia and demonstrate the utility of metabolic profiling in predicting dementia risk. A qualitative summary of all biomarker associations identified in this study is provided in Supplementary Table 7.

### Subgroups II and III, and VaD

**Fig. 6:**
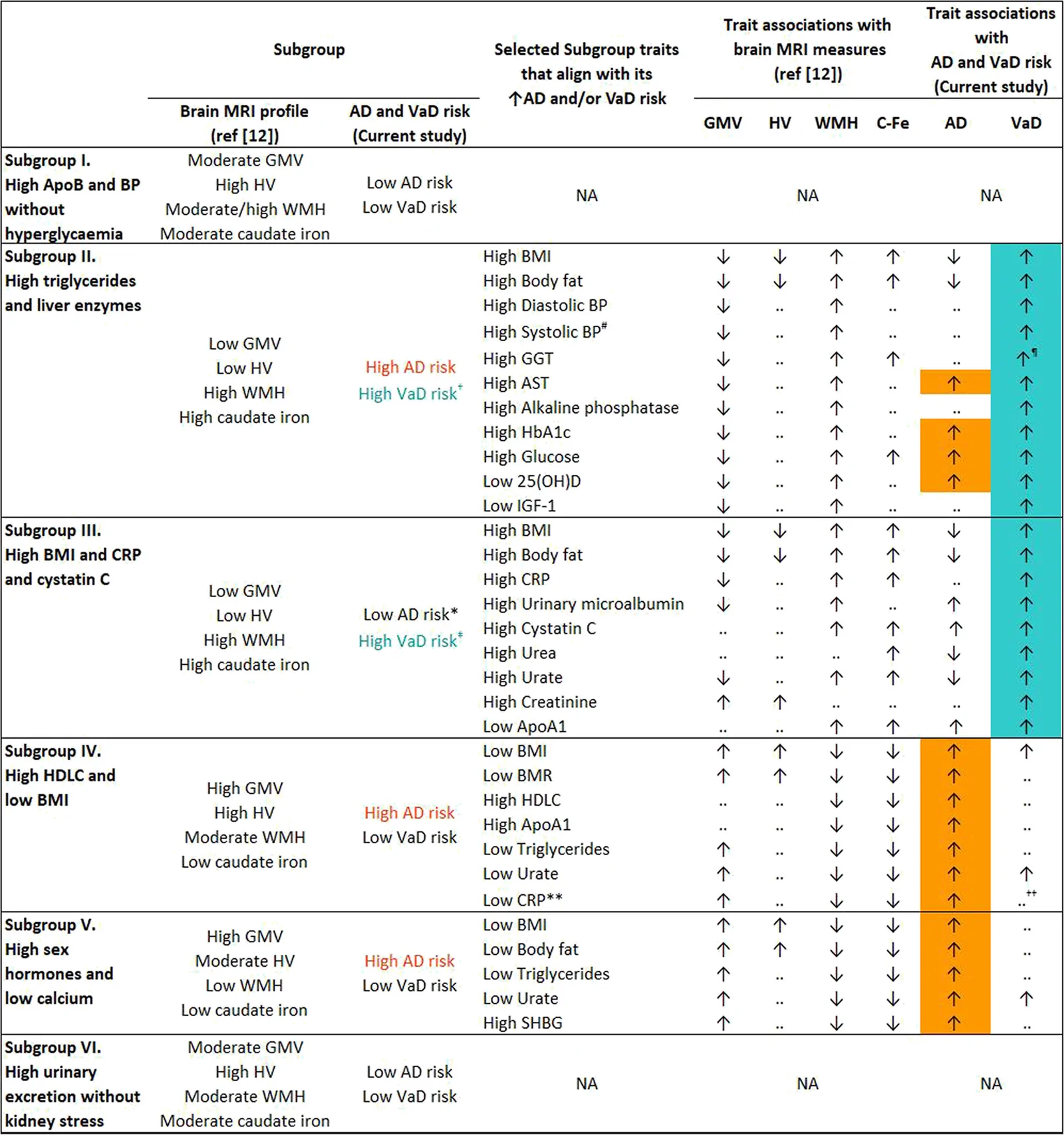
Summary of relationships of metabolic subgroups and their biomarker traits with Alzheimer’s disease and vascular dementia risks, and brain MRI measures. Traits of the six metabolic subgroups are described previously in more detail [10, 12]. The table comprises a summary of the findings of the current study, together with previously reported subgroup and biomarker associations with dementia-related brain MRI measures [12], as indicated. Biomarker associations consistent with overall subgroup risk of AD and/or VaD are highlighted with orange and aqua block colour, respectively. * Lower AD risk in Subgroup III compared to Subgroup IV was evident only after adjustments for socioeconomic and/or lifestyle factors, but not in the basic analysis. † Higher VaD risk in Subgroup II compared to Subgroup IV was only observed in the *APOE*-ε4 noncarrier subpopulation. ‡ Higher VaD risk in Subgroup III compared to Subgroup IV was only observed in *APOE*-ε4 noncarriers and heterozygotes. ¶ The association of higher GGT with higher VaD risk was evident only in *APOE*-ε4 noncarriers. # While high systolic BP is a characteristic trait of subgroup I (which does not show elevated VaD risk) it is shown for Subgroup II here as it is also somewhat high in this subgroup (which does have higher VaD risk). ** While low CRP is not considered a prominent trait of any of the six metabolic subgroups, it is lowest in Subgroup IV. †† The left side of U-shaped CRP–VaD association in the main analysis was attenuated by *APOE*-ε4 adjustment, thus the low CRP trait is indicated as not associating with higher VaD risk here. Two dots (..) indicate that no association was identified. 25(OH)D, 25-hydroxyvitamin D; AD, Alzheimer’s disease; ApoA1, apolipoprotein A1; ApoB, apolipoprotein B; AST, aspartate aminotransferase; BMI, body mass index; BMR, basal metabolic rate; BP, blood pressure; C-Fe, caudate iron; CRP, C-reactive protein; GGT, gamma glutamyltransferase; GMV, grey matter volume; HbA1c, glycated haemoglobin; HDLC, high-density lipoprotein cholesterol; HV, hippocampal volume; IGF-1, insulin-like growth factor 1; MRI, magnetic resonance imaging; NA, not applicable; SHBG, sex hormone binding globulin; WMH, white matter hyperintensities; VaD, vascular dementia.

The two most adipose subgroups (II and III) showed ~ 50–60% higher risk of VaD than lean subgroup IV. Biomarker traits that associated with higher VaD cluster in these two metabolic subgroups, including high adiposity (shared trait), high diastolic blood pressure, hyperglycaemia, high AST, GGT, alkaline phosphatase, and low 25(OH)D (Subgroup II traits), high microalbumin, cystatin C and urate, high CRP, and low ApoA1 (Subgroup III traits). Lower testosterone was also associated with higher VaD risk in males, and although not a characteristic trait of Subgroups II and III, when considering males only, these subgroups have the lowest levels [10]. Consistent with their associations with VaD, Subgroups II and III, and their VaD-associated traits have previously shown associations with adverse brain MRI measures including higher WMH volume, lower grey matter volume (GMV), lower hippocampal volume (HV) and higher caudate iron deposition [12] (Fig. 6, and see Supplementary Fig. 6 for extended information).

Many of these biomarker–VaD associations align with current literature. Diabetes has been linked to VaD risk [20], and may damage cerebral microvasculature via mechanisms including increased blood–brain permeability, altered cerebrovascular morphology, and blood flow dysregulation [21]. Uncontrolled hypertension is also a known risk factor for VaD [22], although, notably we did not see elevated VaD risk in Subgroup I which has characteristically high blood pressure without hyperglycaemia [10]. Having high diastolic blood pressure at a younger age (mid-life) was more risky than in older age, as has been previously reported [23]. Liver enzyme GGT has been linked to higher risk of VaD and all-cause dementia among individuals with diabetes [24] and may implicate oxidative stress in VaD risk. The relationship of AST with higher risk of VaD is not well characterised, although Mendelian randomisation (MR) studies support an adverse effect of genetically predicted AST on microvascular disease-related conditions including hypertension, type 2 diabetes (T2D) and diabetic retinopathy [25–27]. While the inverse association between 25(OH)D and VaD risk may be related to the lowering effect of obesity on 25(OH)D [28], raising 25(OH)D may be beneficial in helping to regulate blood glucose levels [29, 30].

The relationship of Subgroup III traits with higher VaD risk aligns with reported connections of kidney disease with cerebrovascular disease [31] and white matter lesions [31, 32] and may be related to shared vulnerabilities of the kidney and brain to vascular risk factors such as diabetes and hypertension [33], although hyperglycaemia and high blood pressure are not prominent features of Subgroup III. Alternatively, inflammation biomarker CRP may offer a potential link, as high CRP is a Subgroup III trait that was associated with VaD risk, and is a risk factor for kidney disease [34] that also associates with cerebrovascular disease [35].

In sensitivity analyses, Subgroup II and its trait, high GGT, were associated with higher VaD risk only in *APOE*-ε4 noncarriers, while Subgroup III was associated with VaD risk in noncarriers and heterozygotes. This is consistent with previous reports of stronger links between metabolic syndrome or kidney disease and dementia in noncarriers [1, 4], possibly reflecting higher baseline VaD risk in ε4 carriers [36] or allele-specific differences in vulnerability to metabolic changes. For example, the ε2 allele may hasten kidney disease progression [37].

### Diverse subgroups with higher risk of Alzheimer’s disease

In comparison to lean Subgroup IV with high HDLC, Subgroups I and VI had 10–20% lower risk of AD, whereas Subgroups II and V had (high) risk comparable to Subgroup IV. Different subsets of AD-associated biomarkers cluster in the different subgroups with high AD risk (II, IV and V) (Fig. 6). High HbA1c, glucose, AST, and low 25(OH)D are biomarker traits of Subgroup II that associated with higher AD risk. Evidence from MR studies support causal adverse effects of T2D [38–40], and low 25(OH)D [41, 42] on AD risk, and AST may also promote glycaemic dysregulation and diabetes [26], as mentioned above. Our findings support potential benefits of glycaemic control and correction of 25(OH)D deficiency against AD, particularly in overweight individuals with liver stress. Other AD-associated biomarkers were traits that cluster in lean subgroups IV and V, including low BMI, low triglycerides, and low urate (both subgroups), low BMR, high HDLC and ApoA1 (Subgroup IV), high SHBG, and low body fat (Subgroup V) (Fig. 6). MR studies have not supported causal effects of triglycerides, body fat percentage, or SHBG on AD risk [43–45]. Whether HDLC causally affects AD risk remains controversial, with MR studies reporting adverse [46], protective [47–49], and null effects [50–52]. The observed association of low urate with higher AD risk is unlikely to be causal based on MR analyses supporting an adverse effect of urate on AD [53, 54], and may reflect reverse causality [55]. In contrast, MR studies support causal adverse effects of lower BMR [14], and lower lean mass [56] (which, being highly respiratory tissue, is closely related to BMR) on AD risk, which together with our findings potentially highlight this trait as a key AD risk factor among lean individuals. Furthermore, although not a key feature of Subgroup IV, CRP is lowest in this subgroup (Supplementary Fig. 2), and we observed a striking L-shaped association of CRP with AD risk. *APOE*-ε4 is associated with lower CRP levels [17, 19], and accordingly, the risk associated with low CRP was notably attenuated by *APOE*-ε4 adjustment, but not abolished, suggesting that the association is partially but not entirely mediated by *APOE*-ε4. Our findings are consistent with a previously reported association of low CRP levels with higher AD risk among lean individuals (BMI < 25kg/m^2^) [57]. Although low CRP is often interpreted as protective, in the context of low BMI, it could reflect blunted immune responsiveness. However, while plausible protective mechanisms related to the function of the complement system have been proposed [57, 58], evidence of causality from MR studies remains controversial [57–59]. Another possibility is that nutritional stress may activate the hypothalamic–pituitary–adrenal (HPA) axis [60], and elevate levels of cortisol, a potent anti-inflammatory agent [61]. This interpretation is consistent with cortisol’s implication in AD risk [62], and with the lower BMI, higher cortisol, and lower CRP levels observed among *APOE*-ε4 carriers [17, 63].

While Subgroup II is associated with adverse brain MRI traits, lean Subgroups IV and V have MRI traits considered more ‘favourable’; high GMV, moderate/high HV, moderate/low WMH volume, and low caudate iron; and their AD-associated biomarker traits show similar associations [12] (Fig. 6). Neuropathological heterogeneity is recognised in AD, with “hippocampal sparing” being one of four proposed subtypes of AD pathology based on the distribution of tau pathology and brain atrophy [64, 65]. Given that caudate iron is directly correlated with serum iron measures [66], one possibility is that iron deficiency, which is implicated in AD and dementia pathogenesis [67–70], and related to lower muscle mass and sarcopenia [71, 72], may be related to the high AD risk in Subgroups IV and V with ‘favourable’ MRI profiles. Our study therefore suggests that metabolic type may help predict different subtypes of AD pathology and generate new hypotheses around their underlying pathways to disease.

### Insights into opposing adiposity associations with dementia

In our metabolic subgroup dementia analysis, four subtypes—the two leanest and the two most adipose—showed higher risks of dementia outcomes. The two most adipose groups (II and III) had elevated risk of VaD, which was linked to high adiposity, and adverse metabolic traits related to microvascular injury, such as oxidative stress (Subgroup II) and inflammation (Subgroup III). In contrast, AD risk appeared less related to inflammation or oxidative stress, with the associations instead suggesting altered metabolic regulation and substrate availability as risk factors. For example, of the high adiposity subgroups, only Subgroup II showed higher AD risk, with hyperglycaemia appearing to be a main driver, whereas the relatively low AD risk in the other obese subgroup (III), which does not show overt hyperglycaemia, may reflect a relative lack of insulin resistance. The two leanest subgroups (IV and V) also showed elevated AD risk. These subgroups are not characterised by traits of glucose dysregulation, with AD risk linked to their low BMI, BMR and percentage body fat, suggesting limited lean and fat mass and energy reserves. High SHBG, a protein inversely linked to both caloric intake and hepatic lipogenesis [73], further limits bioavailable anabolic hormones (oestradiol and testosterone). Together, these trait associations are consistent with a state of undernutrition or energy deficiency in Subgroups IV and V.

Given that both insulin resistance (Subgroup II) and low lean mass and undernutrition (Subgroups IV and V) are linked to sarcopenia, a recognised risk factor for cognitive decline and dementia [74], this may represent a converging pathway underlying elevated AD risk across these subgroups, despite their opposing adiposities. While the exact mechanisms remain unclear, there is a well-established connection between muscle quality and brain health, with skeletal muscle helping to regulate metabolism and providing substrates and neurotrophic factors that support brain function [74]. Furthermore, elevated AST – an enzyme whose increased serum levels typically indicate liver or skeletal muscle injury – was associated with higher AD risk. Given that ALT, a more liver-specific marker, showed an inverse association, this pattern suggests that the link between AST and AD may primarily reflect muscle rather than liver pathology.

### Strengths and limitations

A strength of our study is the use of a large population and SOM-derived metabolic profiles, which were able to detect differential risks of dementias demonstrating their utility for comparative risk analyses of dementia, and providing information on how dementia-associated biomarkers cluster in the population. The use of both linear and nonlinear modelling in biomarker analyses helped to understand the nature of their different relationships with dementia outcomes. Another strength is the inclusion of sensitivity analyses that revealed additional insight into metabolic relationships with dementia. For example, while the *APOE*-oestradiol-dementia connection is well evidenced in the literature [75–78], it is not well understood and our findings of lower risk of all-cause dementia specifically in *APOE*-ε4 homozygotes, with both AD and VaD showing a similar trend, suggest an intriguing association that warrants further investigation. One possible mechanism, consistent with previous reports, is a differential response to oestradiol by *APOE* genotype [75–77].

Limitations include that while SOM subgroups are based on aggregation of metabolic factors in the population, variation will still exist within each subgroup, with not every individual fitting perfectly in a particular subgroup profile. Some aspects of metabolism may not be captured by our metabolic biomarkers and subgroups. Also, not all biomarker–dementia associations were reflected as metabolic subgroup traits. For example, after adjustment for *APOE*-ε4, low cholesterol levels remained associated with higher risk of dementia, yet none of the subgroups exhibit notably low cholesterol levels [10]. However, the way cholesterol clusters with other traits may provide insight into its relationship with dementia risk at the population level. For example, Subgroup I has characteristically high ApoB, LDLC, total cholesterol, and blood pressure, yet showed low risk of VaD and AD—potentially because of lacking overt inflammation, oxidative stress, hyperglycaemia, or undernutrition. In contrast, while not a defining trait, Subgroup V has the lowest cholesterol levels [10] (Supplementary Fig. 2), and showed higher risk of AD (related to low BMI, triglycerides, urate and higher SHBG); in this context, low LDLC could reflect reduced hepatic output of lipids.

While we have used a prospective study design, further validation is necessary to determine causality of observed associations. The possibility of reverse causality, whereby early subclinical dementia is affecting metabolism, cannot be ruled out. For example, genetic predisposition to AD is reported to lower BMI [79, 80]. While we have made efforts to account for dementia risk factors, we cannot discount possible residual confounding by untested factors, or potential bias from selection or the competing risk of death in our analysis of dementia outcomes [81], however most identified associations have a plausible biological mechanism, suggesting limited impact of this bias. The UKB population is subject to healthy volunteer bias [82] and may not be a true representation of the general population. Our study, and the original metabolic subgroup definition by Mulugeta et al. [10] were performed using white British participants, so validation in other ethnic groups is needed. While metabolic biomarker–dementia associations may be broadly similar, the relative prevalence and defining traits of biomarker clusters may vary across populations due to genetic, physiological, and lifestyle differences. *APOE*-ε4 effects may also vary by ethnicity, although we observed minimal *APOE*-ε4 interactions in our study. Other genetic differences and their potential interactions with these associations cannot be ruled out.

### Conclusion

Our metabolic profiling approach reveals differential risks of dementia outcomes across six diverse profiles, and key biomarkers that may drive these associations. The metabolic diversity underlying dementia risk may help inform precision medicine approaches and development of effective disease-modifying strategies. Regulating blood sugar and blood pressure, and maintaining liver and kidney health and adequate 25(OH)D levels may help reduce the risk of dementia in overweight individuals. Among lean individuals who may be relatively healthy in midlife, strategies to increase BMR and muscle mass may be beneficial.

## Supplementary Information

Below is the link to the electronic supplementary material.

Supplementary file1 (DOCX 4.07 MB)

Supplementary file2 (XLSX 28885 KB)

## Data Availability

The de-identified data generated or analysed as part of this research will be accessible to approved users of the UK Biobank, upon application.

## Abbreviations

95% CI: 95% Confidence interval
A-levels: Advanced Levels
AD: Alzheimer’s disease
AHA: American Heart Association
AIC: Akaike Information Criterion
ALT: Alanine aminotransferase
*APOE*: Apolipoprotein E gene
ApoA1: Apolipoprotein A1
AST: Aspartate aminotransferase
BMR: Basal metabolic rate
BMI: Body mass index
C-Fe: Caudate iron
CRP: C-reactive protein
CSE: Certificate of Secondary Education
GGT: Gamma glutamyltransferase
GMV: Grey matter volume
HbA1c: Glycated haemoglobin
HDLC: High-density lipoprotein cholesterol
HR: Hazard ratio
HV: Hippocampal volume
ICD-10: International Classification of Diseases
LDLC: Low-density lipoprotein cholesterol
LHR: Likelihood ratio
MRI: Magnetic resonance imaging
NVQ: National Vocational Qualification
SHBG: Sex hormone binding globulin
SOM: Self-organising map
T2D: Type 2 diabetes
UKB: UK Biobank
VaD: Vascular dementia
WMH: White matter hyperintensity
