# Correction to: Dementia risk across distinct metabolic profiles in the UK Biobank

**DOI:** 10.1007/s11357-026-02114-0

**Published:** 2026-04-27

**Authors:** Amanda L. Lumsden, Anwar Mulugeta, Elina Hyppönen

**Affiliations:** 1 Australian Centre for Precision Health, Unit of Clinical and Health Sciences, University of South Australia, South Australian Health and Medical Research Institute, P.O. Box 11060, Adelaide, South Australia 5001 Australia; 2 South Australian Health and Medical Research Institute, North Terrace, Adelaide, South Australia 5001 Australia; 3 Department of Pharmacology and Clinical Pharmacy, College of Health Sciences, Addis Ababa University, Addis Ababa, Ethiopia

**Correction to: GeroScience**

10.1007/s11357-025-01970-6

This erratum corrects errors identified in the originally published version of the article.

In the abbreviations section on page 2, the following terms should be deleted:

CHIAG - National Information Governance Board for Health & Social Care in England and Community Health Index Advisory Group

IQR - Interquartile range

MREC - NorthWest Multi-center Research Ethics Committee

In Table 1 on pages 6 - 7, entries under the headings “N (%)” and “Person-years at risk for dementia” have been realigned to the left. The corrected Table 1 is shown below.

In Figure 3 (pages 12–15), the word “Apolipoprotein A1” was missing the letter “t” in the graphs. The corrected Figure 3 is shown below.

On page 20, in the Figure 6 image, the reference number “ref [11]” was incorrect and has been replaced with “ref [12]”. The corrected Figure 6 is shown below.

The Electronic Supplementary Material (available online) has been updated as follows: in the supplementary text and figures file, the typographical error in “Apolipoprotein A1” in Supplementary Figure 3 has been corrected, and occurrences of the gene name APOE have been consistently formatted in italics.

**Table 1:** Incidence rates for dementia, Alzheimer’s disease and vascular dementia by characteristics of the UK Biobank participants.

		N (%)	Person-years at risk for dementia	Incidence rate* (n) for dementia^†^	Incidence rate* (n) for Alzheimer’s disease^†^	Incidence rate* (n) for vascular dementia^†^
Total		308,019	4,126,811	13.2 (5445)	6.3 (2617)	2.9 (1212)
Sex						
Male		143,351 (46.5)	1,899,769	15.2 (2891)	6.7 (1275)	3.8 (725)
Female		164,668 (53.5)	2,227,042	11.5 (2554)	6.0 (1342)	2.2 (487)
	*p*			2.7 × 10^−22^	0.03	6.7 × 10^−20^
Age at baseline						
39 – 49 years		68,384 (22.2)	943,238	0.8 (74)	0.2 (22)	0.1 (9)
50 - 59 years		102,083 (33.1)	1,388,323	4.2 (585)	1.6 (228)	0.8 (110)
60 - 72 years		137,552 (44.7)	1,795,251	26.7 (4786)	13.1 (2367)	6.1(1093)
	*p*			<1.0 × 10^−300^	9.7 × 10^−262^	8.8 × 10^−117^
Education						
None		51,268 (16.6)	670,282	27.8 (1865)	13.8 (927)	6.8 (461)
NVQ/CSE/A-levels		110,268 (35.8)	1,479,898	11.4 (1681)	5.6 (827)	2.6 (379)
Degree/professional		146,483 (47.6)	1,976,631	9.6 (1899)	4.4 (863)	1.9 (372)
	*p*			1.0 × 10^−64^	3.5 × 10^−35^	7.0 × 10^−26^
Employment						
No		23,810 (7.7)	313,489	11.7 (368)	4.6 (144)	3.3 (104)
Retired		107,655 (38.9)	1,404,535	28.5 (3998)	14.1 (1988)	6.2 (881)
1^st^ quartile (Lower working hours)		43,278 (14.1)	586,864	6.7 (395)	3.0 (178)	1.6 (93)
2^nd^ quartile working hours		29,886 (9.7)	408,311	4.7 (192)	2.0 (83)	0.9 (38)
3^rd^ quartile working hours		55,805 (18.1)	763,050	3.7 (285)	1.8 (139)	0.8 (60)
4^th^ quartile (Higher working hours)		47,585 (15.5)	650,563	3.2 (207)	1.3 (85)	0.6 (36)
	*p*			9.6 × 10^−113^	9.9 × 10^−46^	4.9 × 10^−28^
Townsend deprivation index						
Less deprived (below median)		153,958 (50.0)	2,076,406	12.2 (2535)	5.9 (1237)	2.6 (541)
Highly deprived (above median)		154,061 (50.0)	2,050,406	14.2 (2910)	6.7 (1380)	3.2 (671)
	*p*			3.5 × 10^−18^	3.1 × 10^−07^	9.1 × 10^−09^
Smoking						
Never		169,490 (55.0)	2,294,265	11.0 (2515)	5.4 (1238)	2.3 (519)
Ex-smoker		108,128 (35.1)	1,436,014	16.6 (2389)	7.9 (1142)	3.7 (538)
Current		30,401 (9.9)	396,532	13.6 (541)	5.9 (237)	3.9 (155)
	*p*			6.2 × 10^−10^	0.003	5.1 × 10^−08^
Alcohol						
Daily or almost daily		66,688 (21.7)	889,462	13.3 (1183)	5.9 (523)	3.0 (268)
3 or 4 times a week		75,455 (24.5)	1,018,060	10.6 (1082)	5.3 (540)	2.3 (235)
1 or 2 times a week		81,176 (26.3)	1,092,805	11.7 (1283)	5.9 (648)	2.5 (279)
1 to 3 times a month		34,028 (11.0)	457,695	11.8 (538)	5.7 (261)	2.6 (119)
Special occasions only		31,389 (10.2)	416,460	17.0 (708)	8.5 (353)	3.8 (160)
Never		19,283 (6.3)	252,329	25.8 (651)	11.5 (292)	6.0 (151)
	*p*			8.8 × 10^−61^	6.7 × 10^−20^	7.7 × 10^−17^
Physical activity						
None		17,079 (5.6)	221,965	21.0 (467)	8.7 (193)	5.1 (114)
Light/moderate		258,249 (83.8)	3,459,231	13.67 (4724)	6.6 (2292)	3.0 (1049)
Strenuous activity		32,691 (10.6)	445,615	5.7 (254)	3.0 (132)	1.1 (49)
	*p*			6.6 × 10^−34^	2.0 × 10^−06^	6.9 × 10^−12^
Serious illness, injury, or assault to yourself in the last 2 years						
No		281,179 (91.3)	3,783,693	12.6 (4757)	6.2 (2347)	2.7 (1035)
Yes		26,840 (8.3)	343,118	20.1 (688)	7.8 (270)	5.1 (177)
	*p*			1.2 × 10^−21^	0.009	6.1 × 10^−11^
Death of a spouse or partner in last 2 years						
No		303,264 (98.5)	4,064,261	13.0 (5300)	6.3 (2546)	2.9 (1185)
Yes		4,755 (1.5)	62,551	23.2 (145)	11.3 (71)	4.2 (27)
	*p*			6.7 × 10^−4^	0.03	0.54
Healthy diet score						
0 (least healthy)		1,389 (0.5)	18,126	17.1 (31)	4.9 (9)	3.8 (7)
1		43,061 (14.0)	573,364	11.8 (674)	5.1 (292)	2.6 (152)
2		115,022 (37.3)	1,540,803	12.5 (1922)	6.1 (939)	2.8 (437)
3		105,447 (34.2)	1,414,394	13.9 (1963)	6.9 (975)	3.0 (422)
4 (most healthy)		43,100 (14.0)	580,124	14.7 (855)	6.9 (402)	3.3 (194)
	*p*			0.18	0.65	0.59

* Incidence rates are per 10,000 person-years at risk

† p-value is from a likelihood ratio test in a logistic regression model adjusting for age, sex, and assessment centre

A-level, Advanced level; CSE, Certificate of Secondary Education; NVQ, National Vocational Qualification

In Table 2 on page 8, an extraneous horizontal line between “HR” and “95% CI” has been removed, and the text “Adjustment model:” has been aligned with the corresponding model headings. The corrected Table 2 is shown below.

**Table 2:** Metabolic subgroup associations with dementia

	All-cause dementia				Alzheimer’s disease				Vascular dementia			
	HR				HR				HR			
	95%CI				95%CI				95%CI			
Adjustment model:	B	B+S	B+L	B+S+L	B	B+S	B+L	B+S+L	B	B+S	B+L	B+S+L
I. High ApoB and BP without hyperglycaemia	0.97	0.93	0.93	0.93	0.92	0.89	0.90	**0.90**	1.08	1.04	1.05	1.05
	0.89–1.05	0.86–1.02	0.85–1.02	0.84–1.00	0.82–1.04	0.79–1.00	0.79–1.01	**0.78–0.99**	0.89–1.31	0.86–1.26	1.87-1.28	0.85–1.25
II. High triglycerides and liver enzymes	**1.23**	**1.13**	1.09	1.04	1.08	1.00	0.98	0.94	**1.83**	**1.64**	**1.62**	**1.52**
	**1.12–1.35**	**1.03–1.25**	0.99–1.20	0.95–1.15	0.94–1.24	0.87–1.15	0.85–1.12	0.82–1.08	**1.51–2.21**	**1.35–1.98**	**1.33–1.97**	**1.25–1.85**
III. High BMI and CRP and cystatin C	**1.28**	**1.18**	1.09	1.05	1.00	0.93	**0.87**	**0.85**	**1.97**	**1.76**	**1.66**	**1.58**
	**1.17–1.40**	**1.08–1.29**	0.99–1.19	0.96–1.15	0.88–1.14	0.81–1.06	**0.76–1.00**	**0.74–0.97**	**1.65–2.36**	**1.46–2.11**	**1.38–2.00**	**1.31–1.90**
IV. High HDLC and low BMI (Referent)	1.00	1.00	1.00	1.00	1.00	1.00	1.00	1.00	1.00	1.00	1.00	1.00
V. High sex hormones and low calcium	1.06	1.06	1.01	1.02	0.98	0.98	0.94	0.95	1.01	1.00	0.96	0.97
	0.98–1.15	0.97–1.15	0.94–1.10	0.94–1.11	0.87–1.09	0.87–1.10	0.84–1.06	0.85–1.07	0.83–1.21	0.83–1.21	0.80–1.16	0.80–1.17
VI. High urinary excretion without kidney stress	1.00	0.99	0.95	0.95	**0.85**	**0.83**	**0.80**	**0.80**	1.08	1.06	1.03	1.03
	0.91–1.10	0.90–1.08	0.87–1.04	0.87–1.05	**0.74–0.97**	**0.72–0.95**	**0.70–0.92**	**0.70–0.92**	0.88–1.33	0.86–1.30	0.84–1.27	0.83–1.27

Cox proportional hazard regression was used to estimate the hazard ratios (HR) for risk of the dementia outcomes across the metabolic subgroups, in comparison to Subgroup IV. Findings from analyses adjusted for basic covariates (age, sex, and assessment centre), additionally adjusted for socioeconomic (B+S; education, employment status, and Townsend Deprivation Index) or lifestyle covariates (B+L; smoking, alcohol consumption, physical activity, stress-related events in the last two years (yes or no), types of stress events (“serious illness, injury, or assault to yourself”, “death of a spouse or partner”, or “financial difficulties”), and healthy diet), and fully adjusted for basic, socioeconomic, and lifestyle factors (B+S+L) are presented. Associations with p-values < 0.05 are shown in bold. Further p-value information can be found in Supplementary table 4A

Abbreviations: ApoB, apolipoprotein B; BMI, body mass index; BP, blood pressure; CRP, C-reactive protein; HDLC, high density lipoprotein cholesterol

**Fig. 3:**
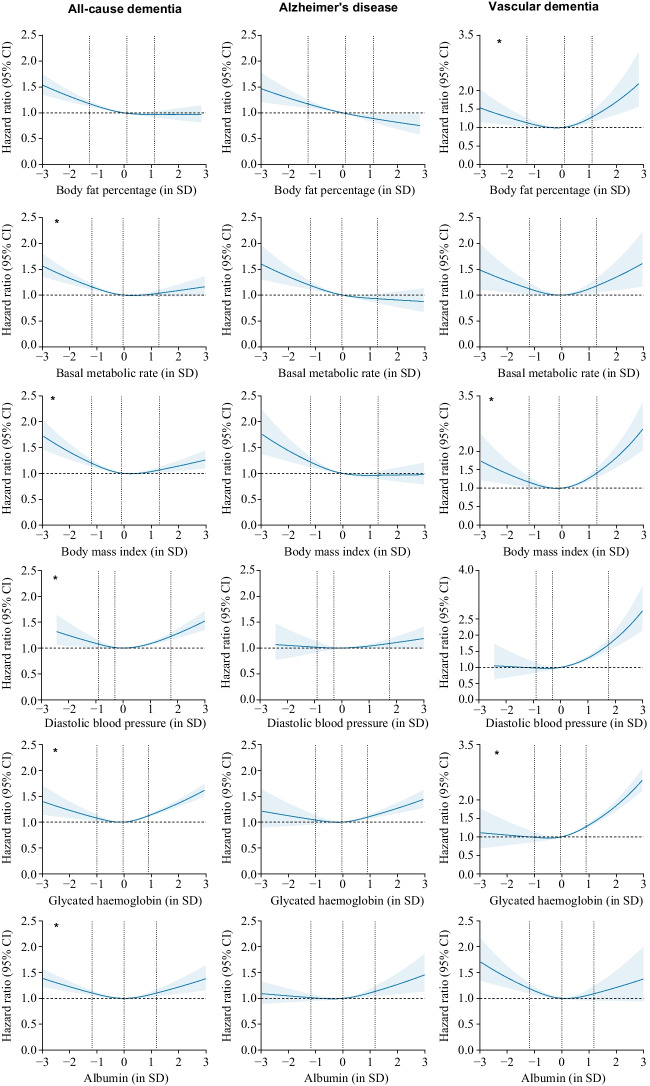

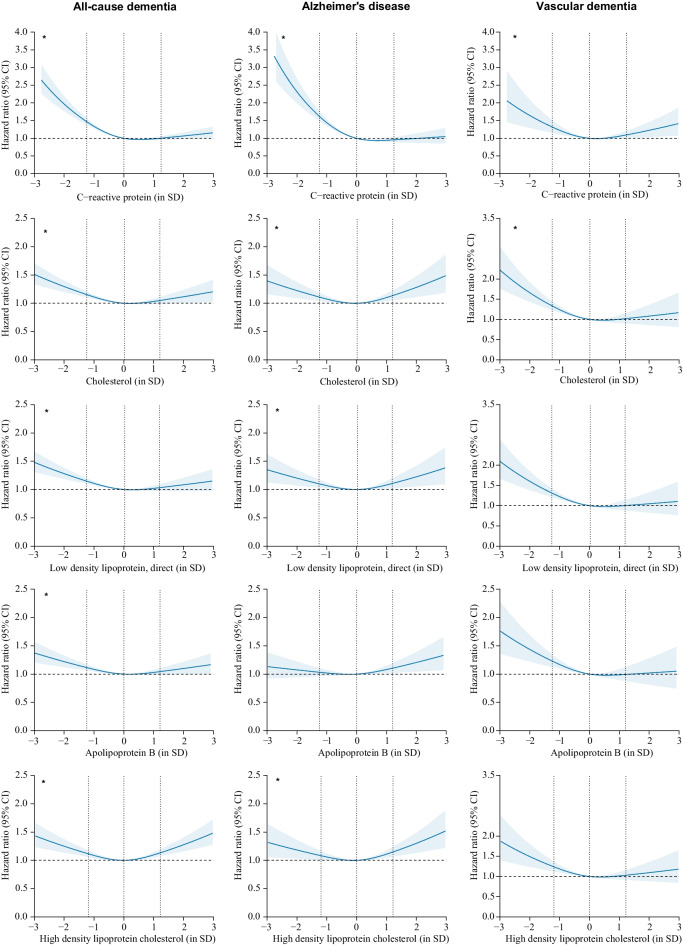

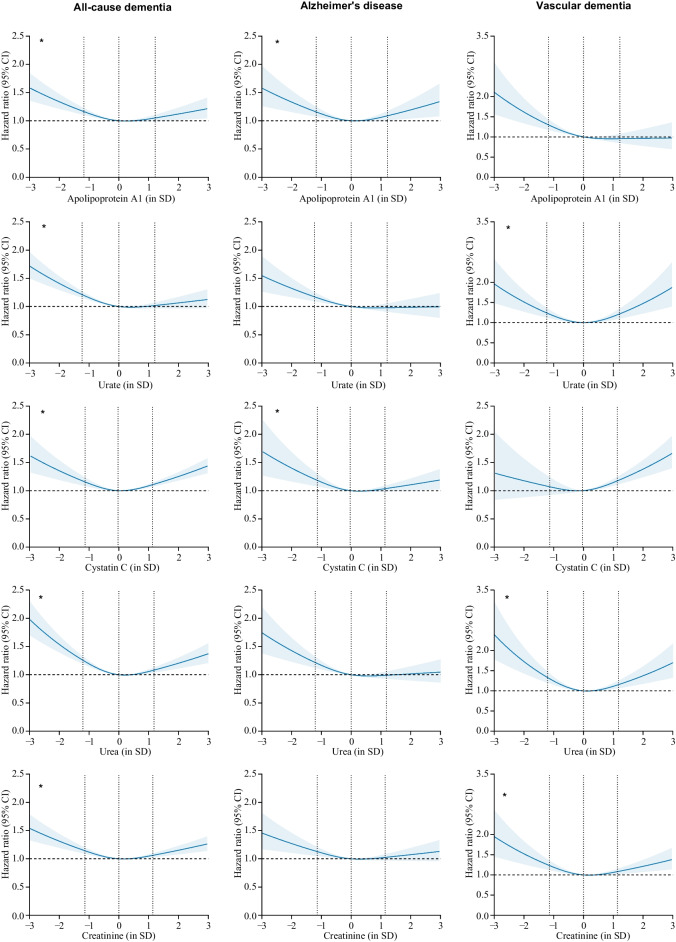

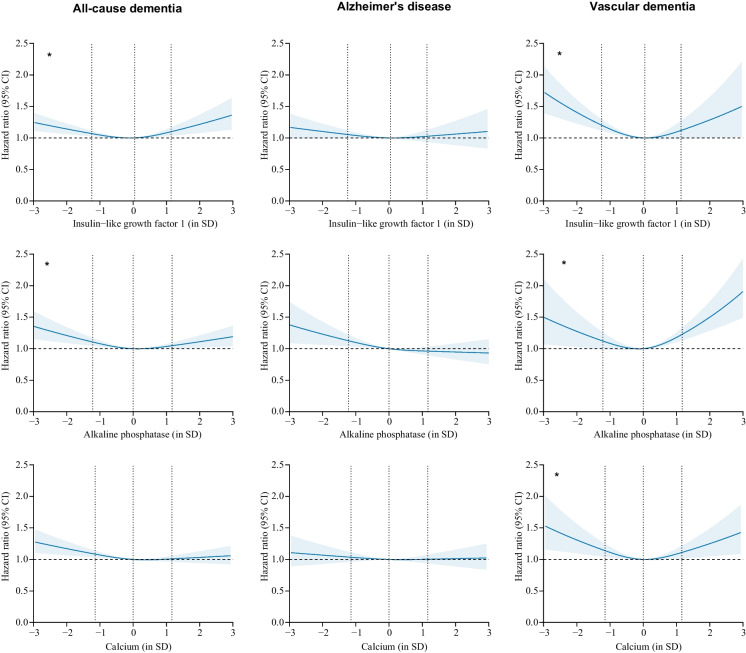
Nonlinear associations between biomarkers and risk of dementia. Restricted cubic spline models fitted for Cox proportional hazard models are shown for all-cause dementia, AD, and VaD, for biomarkers where there was evidence at the Bonferroni-adjusted threshold (pLHR < 0.0013) of a nonlinear relationship for at least one of the dementia outcomes, as indicated with an asterisk (*). Analyses are adjusted for basic (age, sex, and assessment centre), socioeconomic (education, employment status, and Townsend Deprivation Index), and lifestyle (smoking, alcohol consumption, physical activity, stress-related events in the last two years (yes or no), types of stress events (“serious illness, injury, or assault to yourself”, “death of a spouse or partner”, or “financial difficulties”), and healthy diet) covariates. Extended data in Supplementary Fig. 3 shows spline curves for all biomarkers and dementia outcomes. 95% CI, 95% confidence interval; SD, standard deviation.

**Fig. 6:**
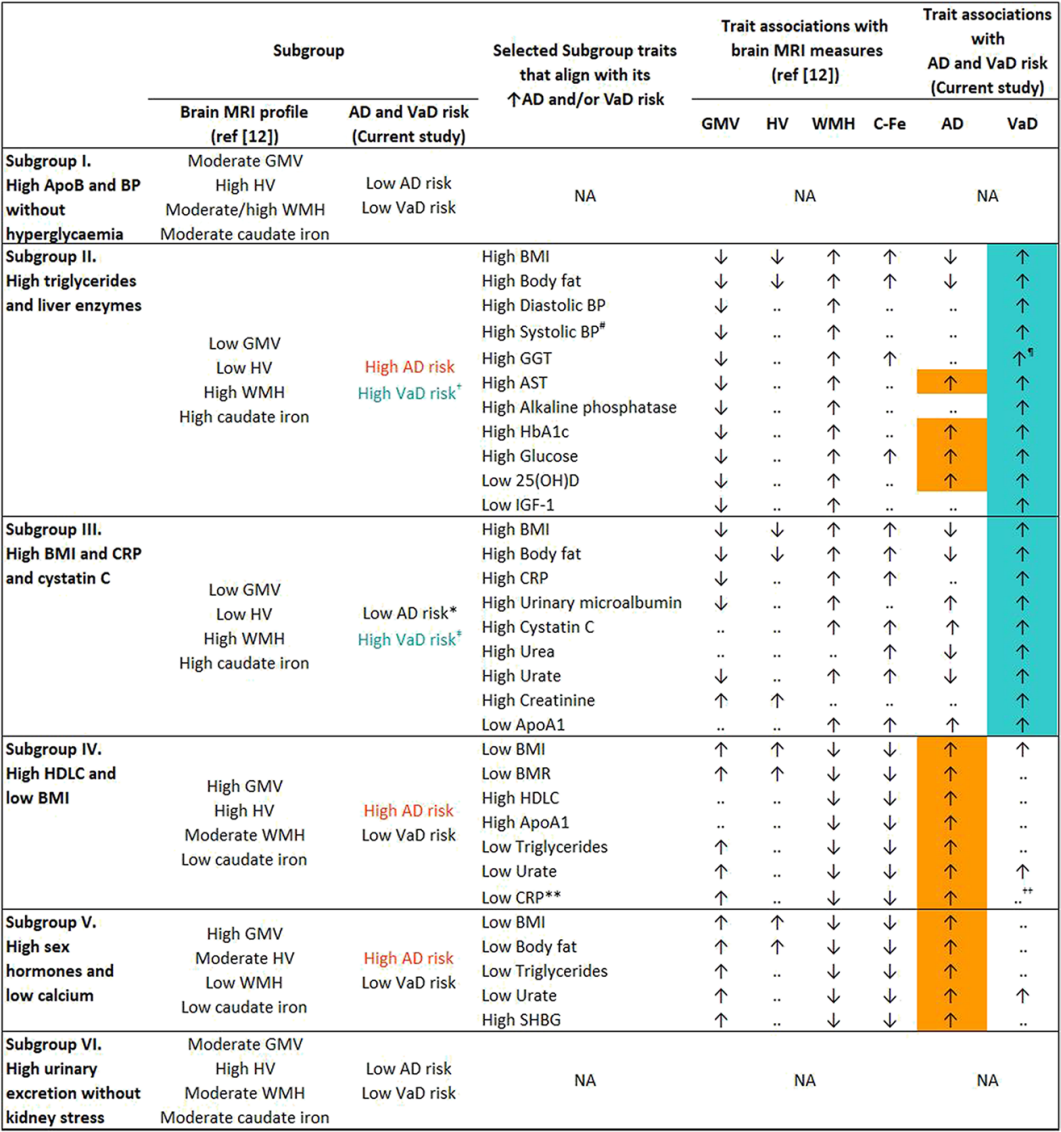
Summary of relationships of metabolic subgroups and their biomarker traits with Alzheimer’s disease and vascular dementia risks, and brain MRI measures. Traits of the six metabolic subgroups are described previously in more detail [10, 12]. The table comprises a summary of the findings of the current study, together with previously reported subgroup and biomarker associations with dementia-related brain MRI measures [12], as indicated. Biomarker associations consistent with overall subgroup risk of AD and/or VaD are highlighted with orange and aqua block colour, respectively. * Lower AD risk in Subgroup III compared to Subgroup IV was evident only after adjustments for socioeconomic and/or lifestyle factors, but not in the basic analysis. † Higher VaD risk in Subgroup II compared to Subgroup IV was only observed in the APOE-ε4 noncarrier subpopulation. ‡ Higher VaD risk in Subgroup III compared to Subgroup IV was only observed in APOE-ε4 noncarriers and heterozygotes. ¶ The association of higher GGT with higher VaD risk was evident only in APOE-ε4 noncarriers. # While high systolic BP is a characteristic trait of subgroup I (which does not show elevated VaD risk) it is shown for Subgroup II here as it is also somewhat high in this subgroup (which does have higher VaD risk). ** While low CRP is not considered a prominent trait of any of the six metabolic subgroups, it is lowest in Subgroup IV. †† The left side of U-shaped CRP–VaD association in the main analysis was attenuated by APOE-ε4 adjustment, thus the low CRP trait is indicated as not associating with higher VaD risk here. Two dots (..) indicate that no association was identified. 25(OH)D, 25-hydroxyvitamin D; AD, Alzheimer’s disease; ApoA1, apolipoprotein A1; ApoB, apolipoprotein B; AST, aspartate aminotransferase; BMI, body mass index; BMR, basal metabolic rate; BP, blood pressure; C-Fe, caudate iron; CRP, C-reactive protein; GGT, gamma glutamyltransferase; GMV, grey matter volume; HbA1c, glycated haemoglobin; HDLC, high-density lipoprotein cholesterol; HV, hippocampal volume; IGF-1, insulin-like growth factor 1; MRI, magnetic resonance imaging; NA, not applicable; SHBG, sex hormone binding globulin; WMH, white matter hyperintensities; VaD, vascular dementia.

## Supplementary Information

Below is the link to the electronic supplementary material.

Supplementary file1 (DOCX 4173 KB)

