# Supplementary material for: Correction to: Dementia risk across distinct metabolic profiles in the UK Biobank
Source: GeroScience. 2026 Apr 27;48(3):5093–102. doi: 10.1007/s11357-026-02114-0 (PMC13356128; doi:10.1007/s11357-026-02114-0)
Supplement: Supplementary file 1 — Supplementary file1 (DOCX 4173 KB) [file 11357_2026_2114_MOESM1_ESM.docx]

# Dementia risk across distinct metabolic profiles in the UK Biobank.

Amanda L. Lumsden^1,2,†^, Anwar Mulugeta^1,2,3,†^, Elina Hyppönen^1,2^

^1^ Australian Centre for Precision Health, Unit of Clinical and Health Sciences, University of South Australia, North Terrace, Adelaide, South Australia 5001, Australia.

^2^ South Australian Health and Medical Research Institute, North Terrace, Adelaide, South Australia 5001, Australia.

^3^ Department of Pharmacology and Clinical Pharmacy, College of Health Sciences, Addis Ababa University, Addis Ababa, Ethiopia.

† These authors contributed equally

Correspondence to: Amanda L. Lumsden

Australian Centre for Precision Health, Unit of Clinical and Health Sciences, University of South Australia, South Australian Health and Medical Research Institute, P.O. Box 11060, Adelaide, South Australia 5001, Australia.

Email: [amanda.lumsden@unisa.edu.au](mailto:amanda.lumsden@unisa.edu.au)

## CONTENTS

| Item | Page |
| --- | --- |
| SUPPLEMENTARY TEXT | 3 |
| SUPPLEMENTARY FIGURE 1 Flowchart of the study population for the analysis. | 4 |
| SUPPLEMENTARY FIGURE 2 Mean metabolic profiles for SOM subgroups normalised by population standard deviation. | 5 |
| SUPPLEMENTARY FIGURE 3 Restricted cubic spline model associations between 39 measures (biochemical and physiological traits) and three dementia outcomes (all-cause dementia, Alzheimer’s disease and vascular dementia). | 6 |
| SUPPLEMENTARY FIGURE 4 Linear biomarker associations with Alzheimer’s disease (AD) and vascular dementia in the main analysis, and after adjustment for *APOE*-ε4 allele number. | 15 |
| SUPPLEMENTARY FIGURE 5 Restricted cubic spline analyses of the relationships of IGF-1 with dementia outcomes using three knot and four knot models; unstratified, and stratified by age. | 16 |
| SUPPLEMENTARY FIGURE 6 Relationships of metabolic subgroups and their biomarker traits with Alzheimer’s disease and vascular dementia risks, and brain MRI measures. | 18 |
| SUPPLEMENTARY REFERENCES | 20 |
|  |  |

## SUPPLEMENTARY TEXT

The subgroup information used in this paper is based on our prior work employing a self-organising map (SOM)-based unsupervised algorithm. SOM, an artificial neural network approach, detects multivariable patterns in complex datasets and allows for visualisation of different subgroups generated from multiple input data. In our previous study, we initially used 54 input variables (including 34 biomarkers, 15 anthropometric measures, and two blood pressure readings). These variables were further reduced to 33 final input variables by converging correlated variables into principal components.

For the individual biomarker analysis, we included 34 biochemical (biomarkers) and five physiological traits, which all except basal metabolic rate (BMR), were considered in our prior self-organising map (SOM)-based subgroup analysis. The 34 biomarkers were classified in to six domains as per the UK Biobank biomarker assay group. These included diabetes biomarkers (glucose and glycated haemoglobin (HbA1c)), liver biomarkers (alanine aminotransferase (ALT), albumin, aspartate aminotransferase (AST), gamma glutamyltransferase (GGT), direct bilirubin and total bilirubin), cardiovascular biomarkers (total cholesterol, low density lipoprotein cholesterol (LDLC), high density lipoprotein cholesterol (HDLC), triglycerides, apolipoprotein A1 (ApoA1), apolipoprotein B (ApoB), lipoprotein A (Lp(A)), and C-reactive protein (CRP)), kidney biomarkers (serum creatinine, creatinine in urine, cystatin C, phosphate, total protein, urate, urea, potassium in urine, sodium in urine, microalbumin in urine), cancer and growth biomarkers (oestradiol, testosterone, sex hormone binding globulin (SHBG) and insulin-like growth factor-1 (IGF-1)), and bone and joint biomarkers (alkaline phosphatase, calcium and 25-hydroxyvitamin D (25(OH)D)). Serum biomarkers obtained from aliquot three were excluded from the analysis due to poor quality of the aliquot [1]. For three biomarkers (oestradiol, rheumatoid factor and microalbumin) with high missing data due to naturally low/undetectable values (>70%), we replaced the missing values with half the value of the lower limit of detection of each biomarker for those individuals who had undergone the test, using values of 36.5 pmol/L for oestradiol, 5 IU/ml for rheumatoid factor, and 3.35 mg/L for microalbumin. In addition to four physiological measures including body mass index (BMI; defined as weight over height (in m) squared), body fat percentage, and diastolic and systolic blood pressure measures), we also included BMR in our analyses.

## SUPPLEMENTARY FIGURE 1


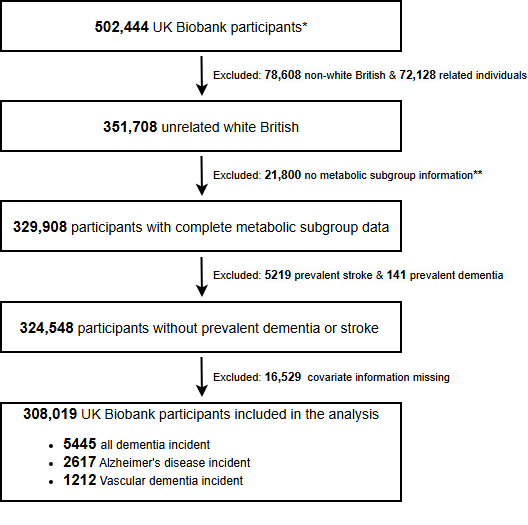


**SUPPLEMENTARY FIGURE 1** **Flowchart of the study population for the analysis**

* Actively consented participants

** This subgroup information was from our prior study [2] which further excluded individuals with no genetic information (*n* = 14,236), biomarkers information from aliquot 3 or 4 (*n* = 7562), and those with missing information for all biomarkers (*n* = 2).

## SUPPLEMENTARY FIGURE 2


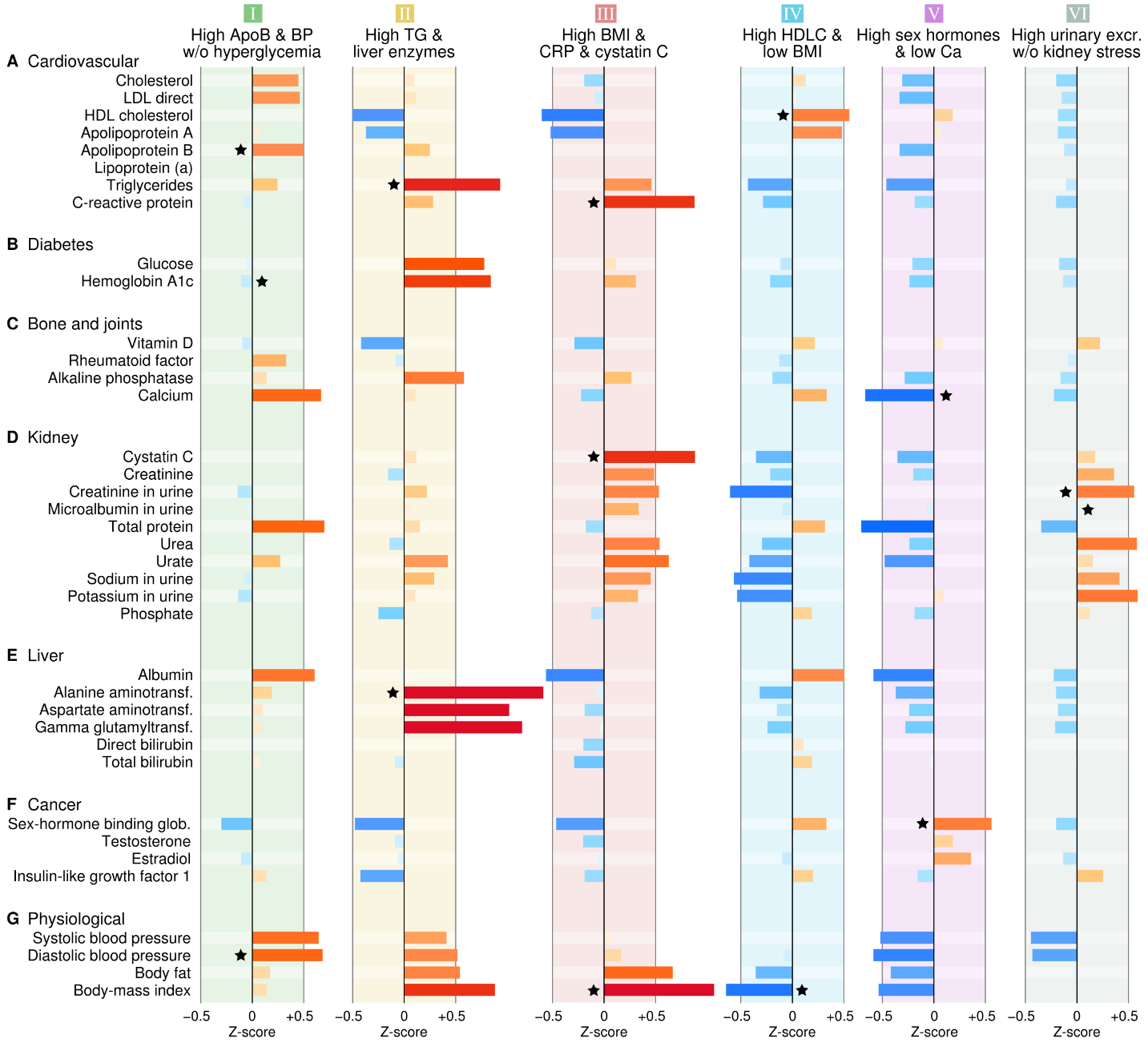


**SUPPLEMENTARY FIGURE 2 Mean metabolic profiles for SOM subgroups normalised by population standard deviation.** The bars are coloured according to the direction and magnitude of the deviation from the population mean. The black stars indicate characteristic features that were selected for simplified naming of the subgroups. *Reproduced from Mulugeta et al.,* Scientific Reports*, 2022 [2], under the terms of the Creative Commons Attribution 4.0 International License (*<http://creativecommons.org/licenses/by/4.0/>*).*

## SUPPLEMENTARY FIGURE 3


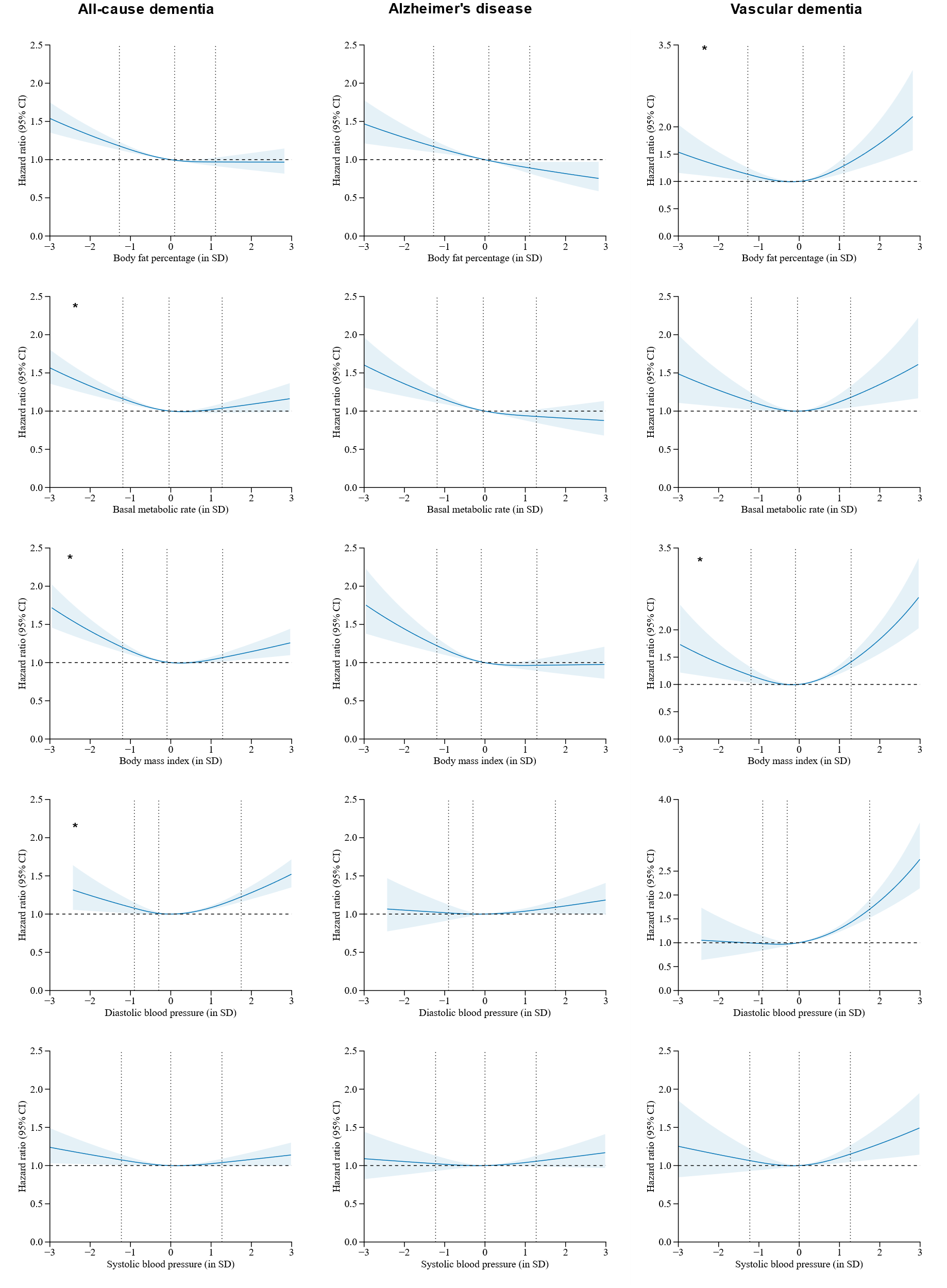


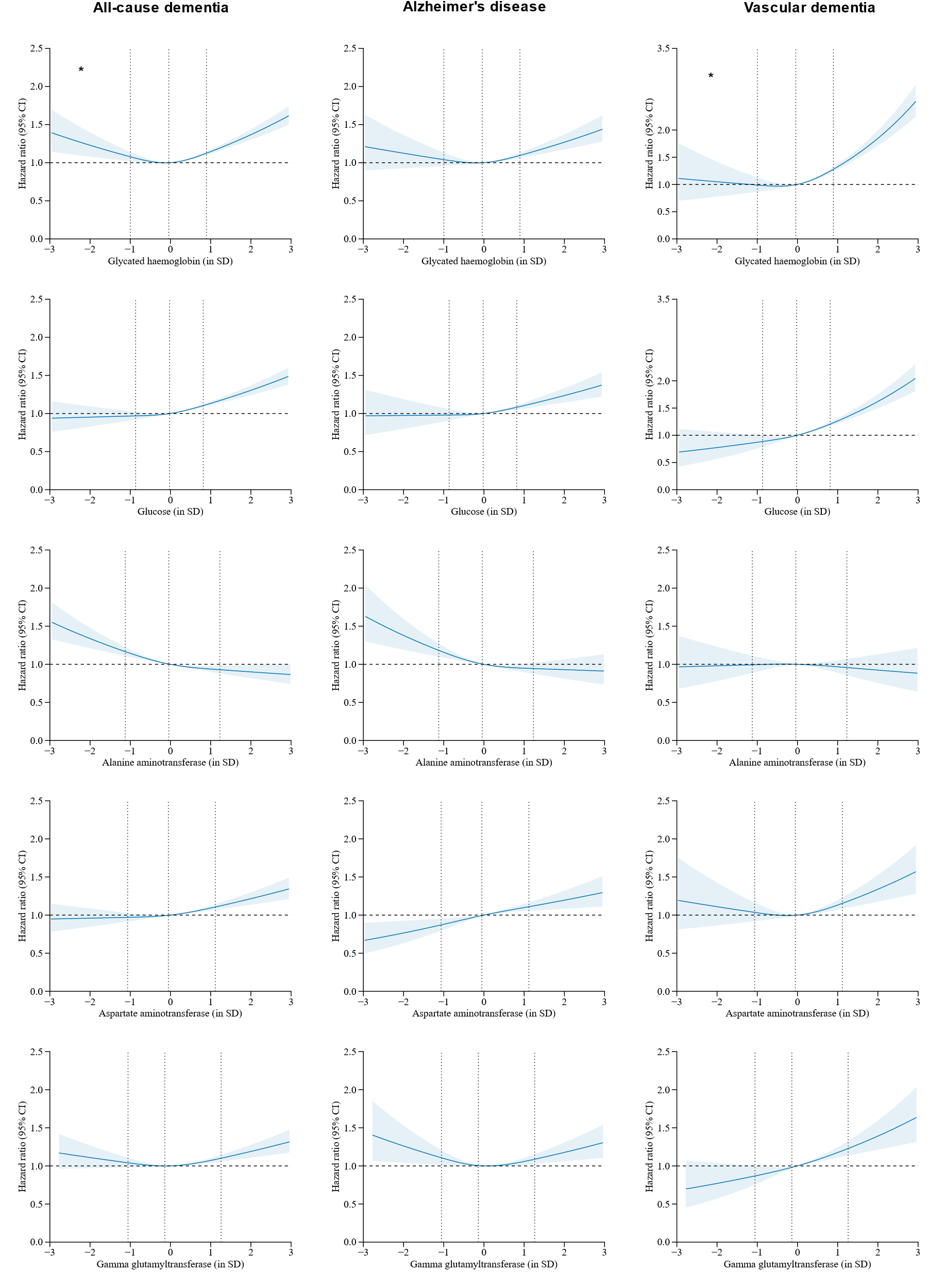


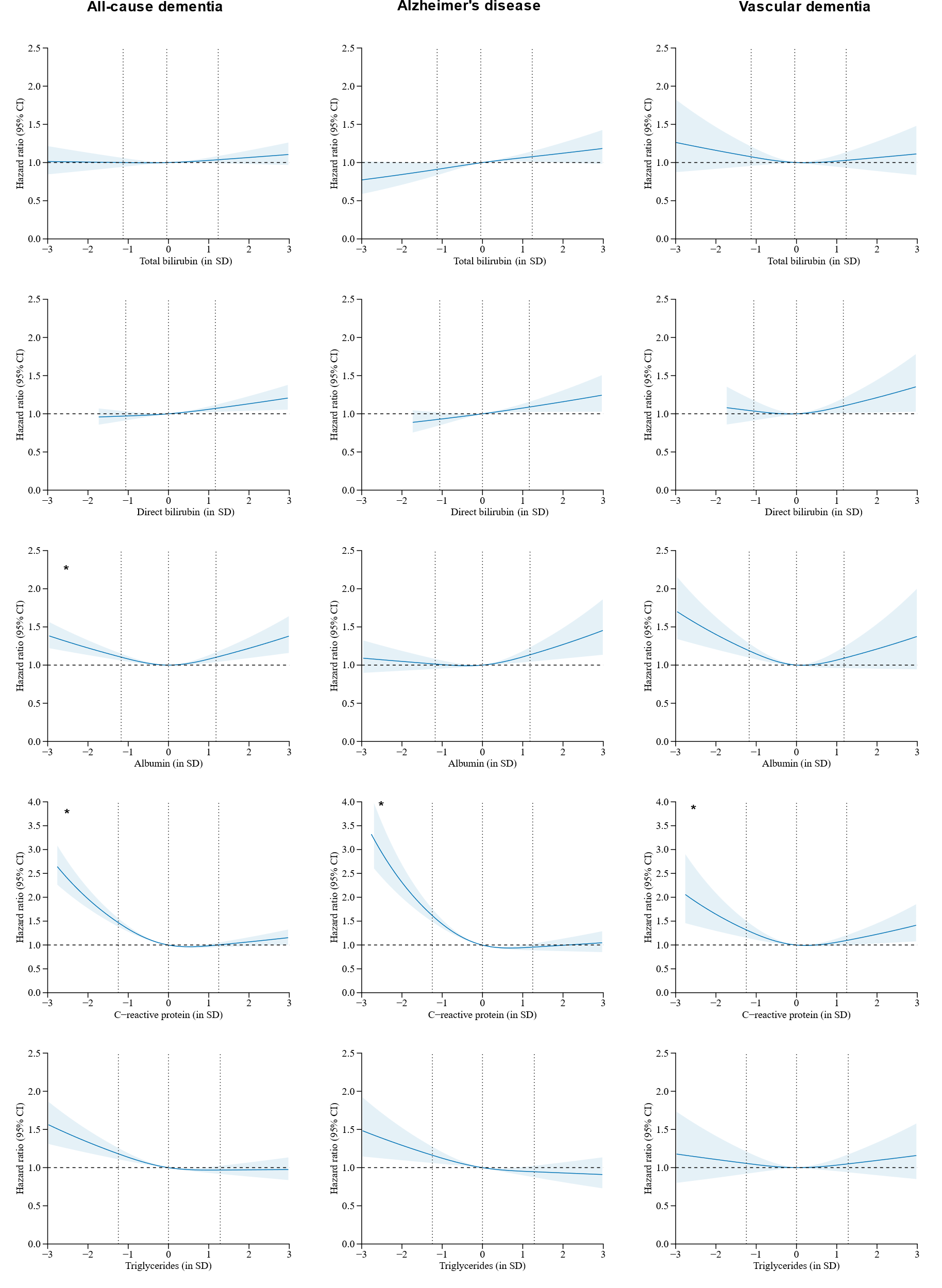


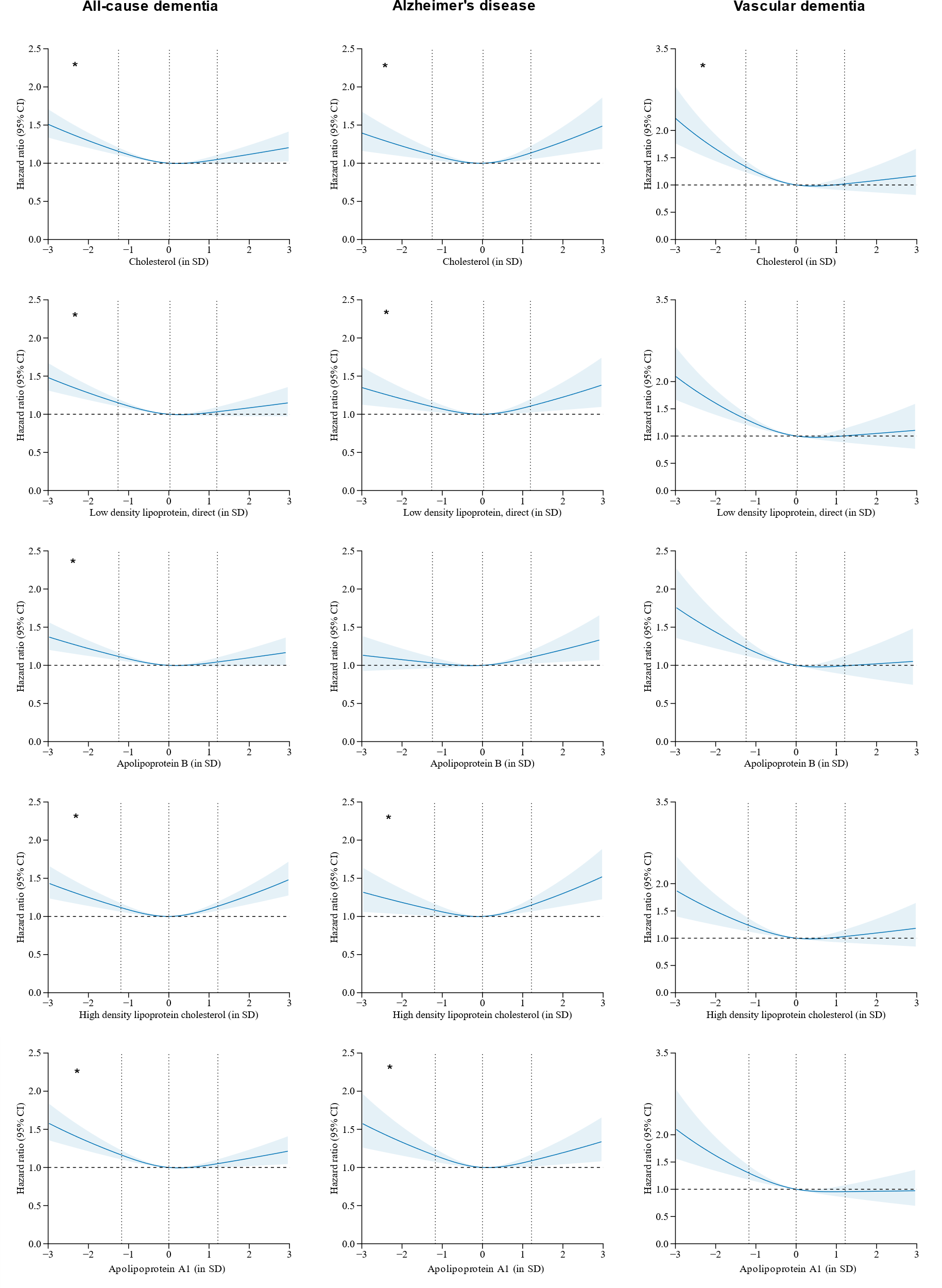


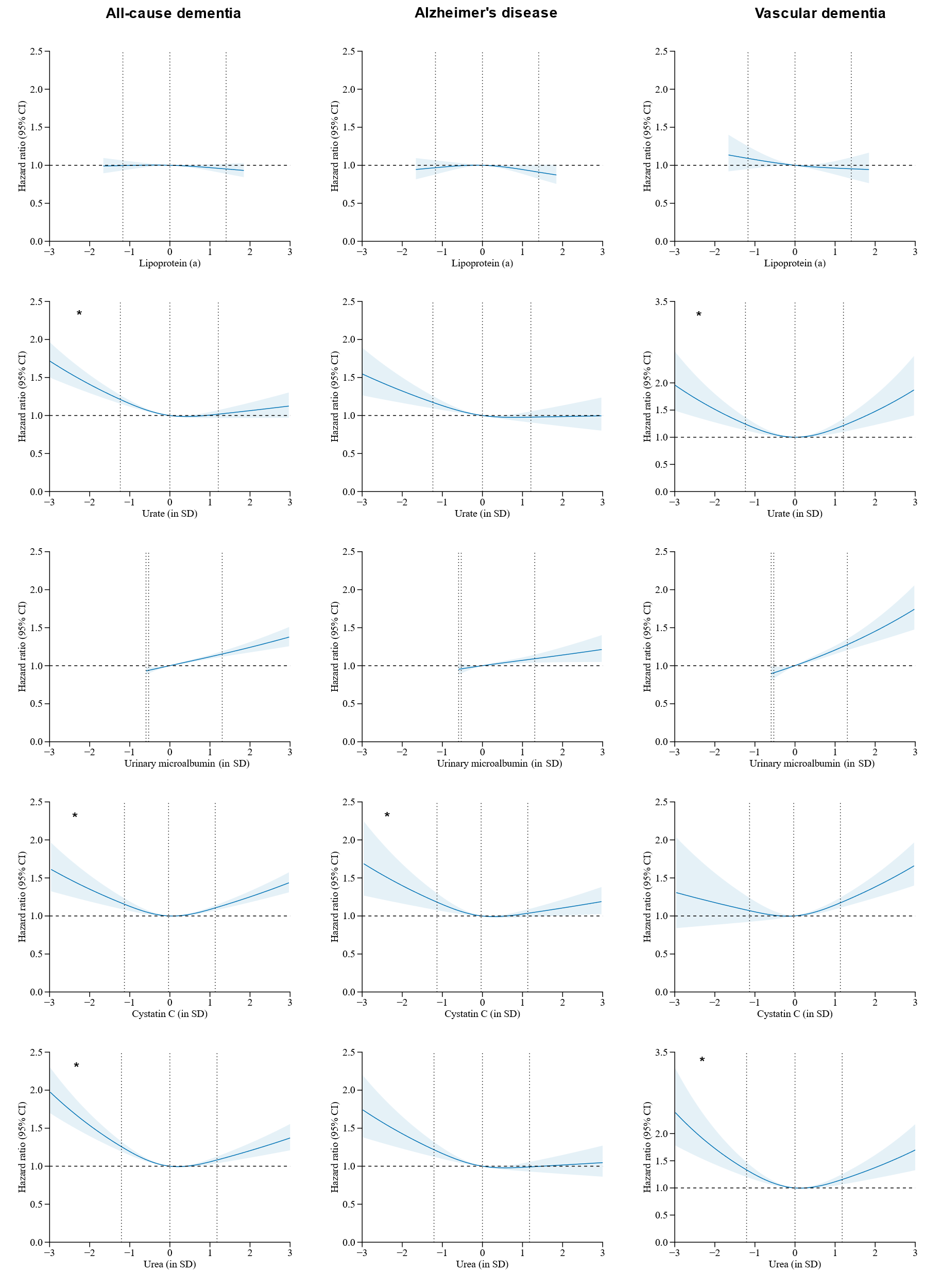


**
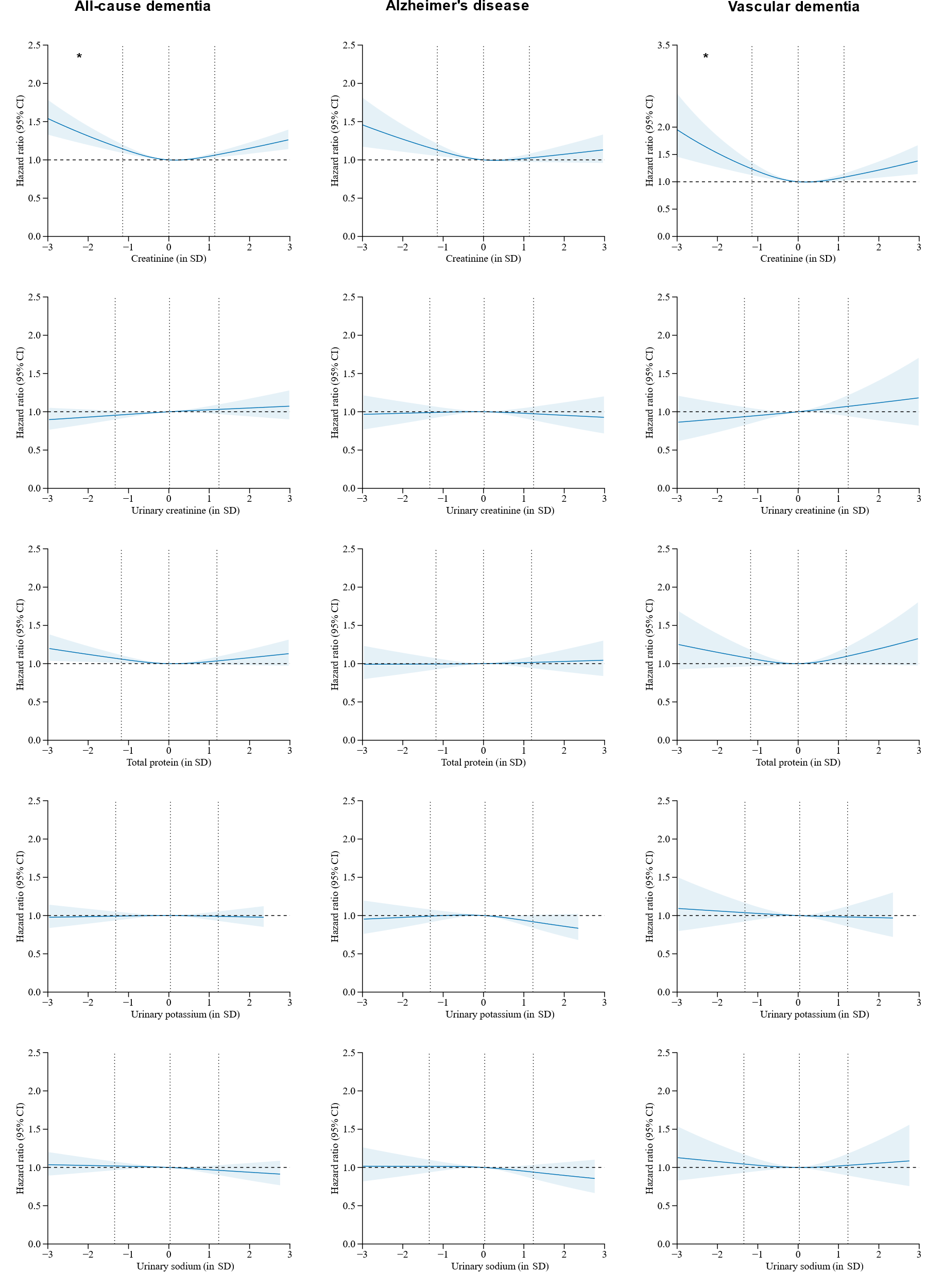
**

**
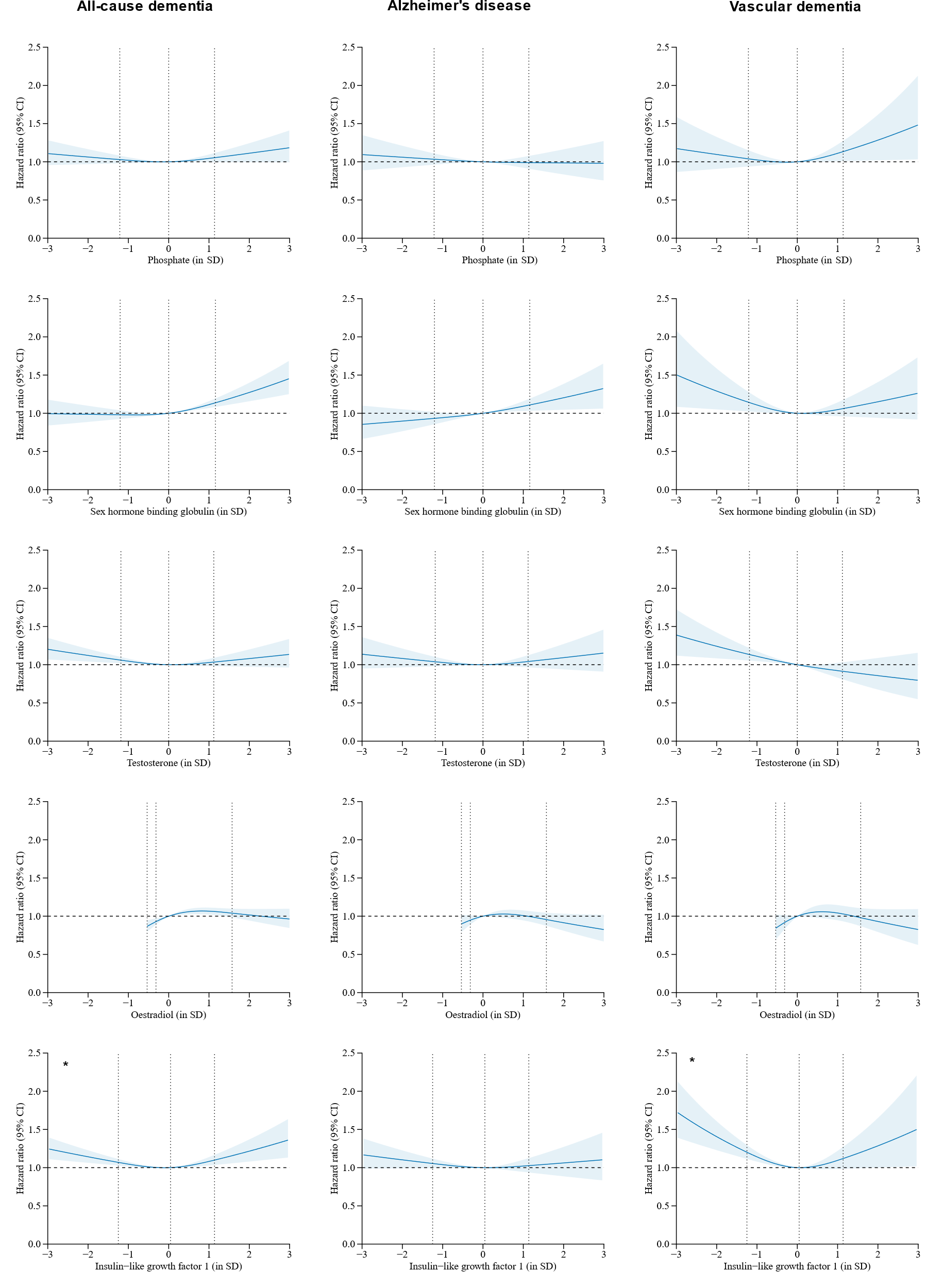
**

**
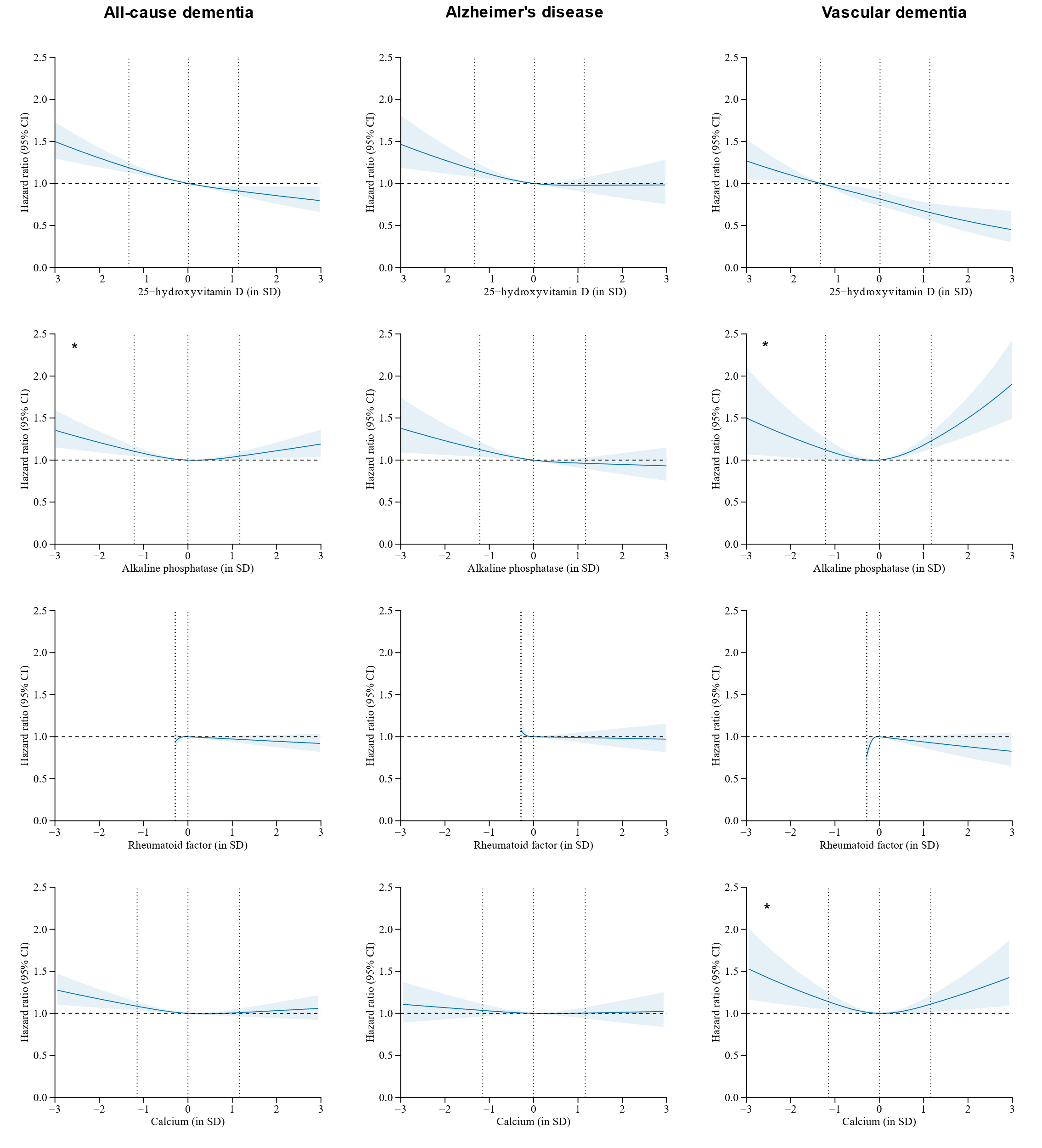
**

**SUPPLEMENTARY FIGURE 3 Restricted cubic spline model associations between 39 measures (biochemical and physiological traits) and three dementia outcomes (all-cause dementia, Alzheimer’s disease and vascular dementia).** Restricted cubic spline models are fitted for Cox proportional hazard models, and analyses are adjusted for basic (age, sex, and assessment centre), socioeconomic (education, employment status, and Townsend Deprivation Index), and lifestyle (smoking, alcohol consumption, physical activity, stress-related events in the last two years (yes or no), types of stress events (“serious illness, injury, or assault to yourself”, “death of a spouse or partner”, or “financial difficulties”), and healthy diet) covariates. Systolic and diastolic blood pressure measures were additionally adjusted for use of blood pressure-lowering medications. An asterisk (*) indicates associations where there is evidence at the Bonferroni-adjusted threshold (*p*_LHR_ <0.0013) nonlinear model is superior to linear modelling by likelihood ratio test. 95% CI, 95% confidence interval; SD, standard deviation.

## SUPPLEMENTARY FIGURE 4


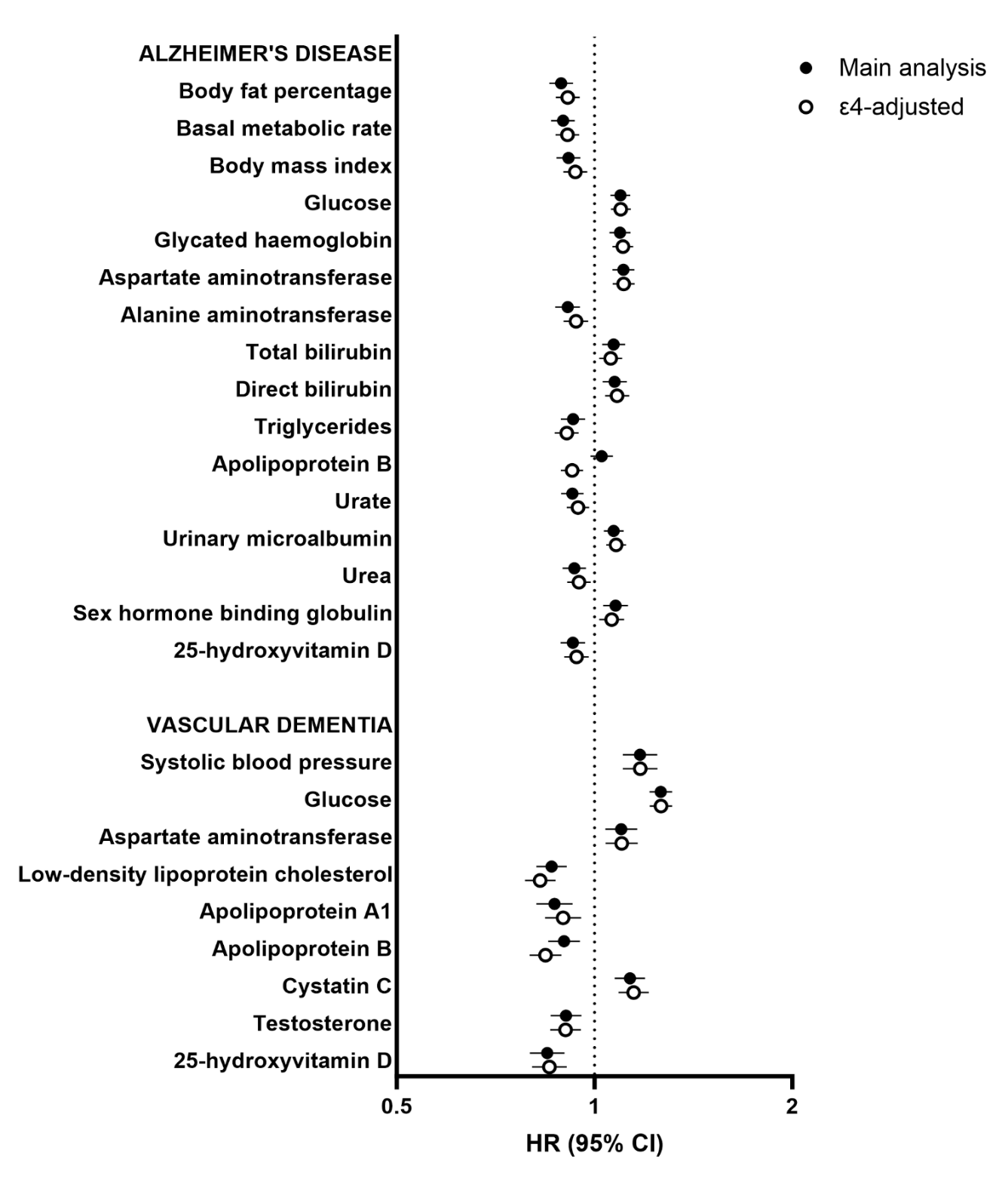


**SUPPLEMENTARY FIGURE 4 Linear biomarker associations with Alzheimer’s disease (AD) and vascular dementia in the main analysis, and after adjustment for *APOE*-ε4 allele number.** *APOE*-ε4 adjustment exposed an inverse association between ApoB and AD. All analyses are adjusted for basic (age, sex, assessment centre), socioeconomic (education, Townsend Deprivation Index, and employment) and lifestyle factors (smoking, alcohol consumption, physical activity, stress-related events in the last two years (yes or no), types of stress events (“serious illness, injury, or assault to yourself”, “death of a spouse or partner”, or “financial difficulties”), and healthy diet). Systolic and diastolic blood pressure measures were additionally adjusted for use of blood pressure-lowering medications. HR Hazard ratio; 95% CI, 95% confidence interval.

## SUPPLEMENTARY FIGURE 5


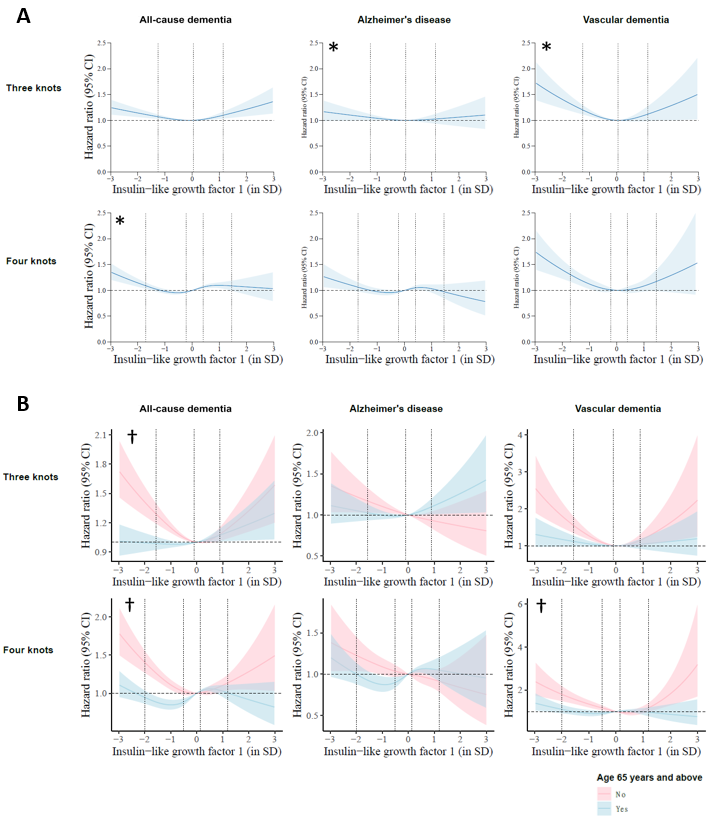


**SUPPLEMENTARY FIGURE 5 Restricted cubic spline analyses of the relationships of IGF-1 with dementia outcomes using three knot and four knot models; unstratified, and stratified by age.** Restricted cubic spline models are fitted for Cox proportional hazard models, and analyses are adjusted for basic (age, sex, and assessment centre), socioeconomic (education, employment status, and Townsend Deprivation Index), and lifestyle (smoking, alcohol consumption, physical activity, stress-related events in the last two years (yes or no), types of stress events (“serious illness, injury, or assault to yourself”, “death of a spouse or partner”, or “financial difficulties”), and healthy diet) covariates. **A)** Unstratified splines showing the relationship between IGF-1 and dementia outcomes, using models of three knots (placed at the 10th, 50th, and 90th percentiles) and four knots (5th, 35th, 65th, and 95th percentiles). The asterisk (*) indicates the model with the optimal number of knots for each IGF-1–outcome relationship, based on comparison of Akaike Information Criterion (AIC) (see **Supplementary table 2**). Note that IGF-1–dementia is the only association that was more optimal with 4 knots. **B)** Age-stratified splines showing the relationship between IGF1 and dementia outcomes among those aged under 65 years (red), versus those aged 65 years and above (blue), in three and four-knot models. The dagger symbol (†) indicates the models that show an age interaction (p<0.0013). A U-shaped association of IGF-1 with dementia was seen in those under 65 years of age, while a more complex pattern that was mostly crossing the null (and more unstable between three and four knot models), was observed in those aged 65 and over. 95% CI, 95% confidence interval; SD, standard deviation.

## SUPPLEMENTARY FIGURE 6

**SUPPLEMENTARY FIGURE 6 Relationships of metabolic subgroups and their biomarker traits with Alzheimer’s disease and vascular dementia risks, and brain MRI measures.** Traits of the six metabolic subgroups are described previously in more detail [2]. The table comprises a summary of the relationships of the metabolic subgroups and their characteristic traits, with Alzheimer’s disease and vascular dementia (from the current study), and with dementia-related brain MRI measures previously reported [3], as indicated. Subgroup traits that are associated with increased risk of AD and/or VaD are highlighted in pale yellow.

* Lower AD risk in Subgroup III compared to Subgroup IV was evident only after adjustments for socioeconomic and/or lifestyle factors, but not in the basic analysis.

† Higher VaD risk in Subgroup II compared to Subgroup IV was only observed in the *APOE*-ε4 noncarrier subpopulation.

‡ Higher VaD risk in Subgroup III compared to Subgroup IV was only observed in *APOE*-ε4 noncarriers and heterozygotes

§ Right side of U-shaped LDLC-AD association in the main analysis was attenuated by *APOE*-ε4 adjustment, thus the high LDLC trait is indicated as not associating with AD here.

¶ The linear association of high ApoB with lower AD risk was only evident after *APOE*-ε4 adjustment.

# The left side of U-shaped HDLC–AD association in the main analysis was attenuated by *APOE*-ε4 adjustment, thus the low HDLC trait is indicated as not associating with higher AD risk here.

** Suggestive evidence supported an association of higher oestradiol with lower dementia risk among *APOE*-ε4 homozygotes

†† The association of higher GGT with higher VaD risk was evident only in *APOE*-ε4 noncarriers

‡‡ While high systolic BP is a characteristic trait of Subgroup I (which does not show elevated VaD risk), it is also somewhat high in Subgroup II (which does have higher VaD risk)

§§ While low CRP is not considered a prominent trait of any of the six metabolic subgroups, it is lowest in Subgroup IV

¶¶ The left side of U-shaped CRP–VaD association in the main analysis was attenuated by *APOE*-ε4 adjustment, thus the low CRP trait is indicated as not associating with higher VaD risk here.

.. Association not identified

25(OH)D, 25-hydroxyvitamin D; AD, Alzheimer’s disease; ApoA1, apolipoprotein A1; ApoB, apolipoprotein B; AST, aspartate aminotransferase; BMI, body mass index; BMR, basal metabolic rate; BP, blood pressure; C-Fe, caudate iron; CRP, C-reactive protein; GGT, gamma glutamyltransferase; GMV, grey matter volume; HbA1c, glycated haemoglobin; HDLC, high-density lipoprotein cholesterol; HV, hippocampal volume; IGF-1, insulin-like growth factor 1; LDLC, low-density lipoprotein cholesterol, MRI, magnetic resonance imaging; SHBG, sex hormone binding globulin; WMH, white matter hyperintensities; WMV, white matter volume, VaD, vascular dementia.

## SUPPLEMENTARY REFERENCES

1. Daniel Fry, et al. UK Biobank biomarker project: Companion document to accompany serum biomarker data 2019 [cited 2019 29 May]; Available from: https://kirov.psycm.cf.ac.uk/Biochemical%20markers/BB%20document_serum_biochemistry.pdf.

2. Mulugeta, A., et al., Cross-sectional metabolic subgroups and 10-year follow-up of cardiometabolic multimorbidity in the UK Biobank. *Sci Rep*, 2022. **12**(1): p. 8590.

3. Lumsden, A.L., et al., Metabolic profile-based subgroups can identify differences in brain volumes and brain iron deposition. *Diabetes Obes Metab*, 2023. **25**(1): p. 121-131.
